# Proprotein Convertase Subtilisin/Kexin Type 9 (PCSK9): The Multifaceted Biology, Diseases, and Pharmaceutical Interventions

**DOI:** 10.1002/mco2.70451

**Published:** 2025-11-02

**Authors:** Jia Kuang, Lei Hao, Meibiao Zhang, Zhao Yang

**Affiliations:** ^1^ Department of Neurology The Affiliated Yongchuan Hospital of Chongqing Medical University Chongqing China; ^2^ Department of Neurology The First Affiliated Hospital of Chongqing Medical University Chongqing China

**Keywords:** PCSK9, LDL‐C, ischemic stroke, intervention strategies, precision therapy

## Abstract

Ischemic stroke remains a leading cause of global disability and death. Proprotein convertase subtilisin/kexin type 9 (PCSK9) inhibitors have emerged as potent lipid‐lowering agents with expanding therapeutic potential. Beyond robust low‐density lipoprotein cholesterol reduction, accumulating evidence suggests these drugs may confer benefits in ischemic stroke prevention and management. However, challenges regarding accessibility, real‐world efficacy, and integration into combination therapies persist, necessitating a comprehensive evidence synthesis. This review systematically consolidates the molecular mechanisms of PCSK9 inhibition and classifies current inhibitors. We delineate recent preclinical advances underscoring their neuroprotective and vasculoprotective effects, alongside critical findings from major clinical trials. These developments highlight promising avenues for both secondary prevention and acute‐phase treatment strategies. Collectively, this synthesis establishes a foundational framework for positioning PCSK9 inhibitors as transformative agents in stroke therapeutics and paves the way for precision neurovascular medicine.

## Graphical abstract

Summary of PCSK9 inhibitor mechanisms. PCSK9 inhibitors (PCSK9‐i) primarily lower LDL‐cholesterol (LDL‐C) by blocking PCSK9‐mediated degradation of hepatic LDL receptors (LDLR), enhancing LDL clearance. Beyond LDLR‐dependent effects, PCSK9‐i exert multiple benefits via LDLR‐independent pathways. These include anti‐inflammatory effects, antioxidant actions, and improved endothelial function. Additionally, PCSK9‐i may modulate immune responses, thrombosis, and metabolic pathways. They also influence plaque stability by decreasing smooth muscle cell proliferation and oxidative stress.

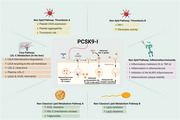

## 1 Introduction

Proprotein convertase subtilisin/kexin type 9 (PCSK9) is a pivotal serine protease primarily synthesized in the liver, playing a critical role in cholesterol metabolism by promoting the degradation of hepatic low‐density lipoprotein receptors (LDLRs) [1, 2]. Structurally, PCSK9 consists of a signal peptide, a prodomain, a catalytic subunit, and a C‐terminal domain, with its function tightly regulated by autocatalytic processing. Since its initial discovery in 2003 through genetic linkage analyses, PCSK9 has rapidly emerged as a central player in lipid homeostasis. Landmark studies, including the identification of gain‐of‐function mutations associated with familial hypercholesterolemia (FH) and loss‐of‐function (LOF) variants linked to hypocholesterolemia and reduced cardiovascular risk, established its therapeutic significance, ultimately culminating in the development of monoclonal antibody inhibitors.

Beyond its canonical role in LDL cholesterol regulation, accumulating evidence underscores the involvement of PCSK9 in extrahepatic pathological processes—particularly in cerebrovascular and neurovascular diseases. PCSK9 is expressed in vascular smooth muscle cells (VSMCs) and endothelial cells, where it exacerbates inflammatory responses, promotes atherosclerotic plaque progression and instability, and enhances platelet activation and thrombogenesis. These pleiotropic mechanisms position PCSK9 at the intersection of dyslipidemia, inflammation, and thrombosis—key drivers of ischemic stroke, which remains a leading cause of mortality and long‐term disability worldwide [3, 4, 5].

Despite the established efficacy of statins, significant residual cardiovascular risk persists, underscoring the need for novel therapeutic strategies. PCSK9 inhibitors (PCSK9‐i), which achieve profound and sustained LDL cholesterol (LDL‐C) reduction, have demonstrated promising pleiotropic benefits including plaque stabilization, endothelial protection, and antithrombotic effects [6]. Nevertheless, current clinical management has yet to fully embrace a mechanism‐based, precision medicine approach for PCSK9 modulation. Important challenges—such as treatment accessibility, real‐world effectiveness, optimal timing in acute settings, and integration with combination therapies—remain unresolved [7, 8, 9, 10].

This review seeks to synthesize contemporary evidence on the therapeutic potential of PCSK9 inhibition in ischemic stroke, spanning from molecular mechanisms to clinical applications. We begin by delineating the biochemical and structural features of PCSK9 and summarizing milestone discoveries in its research trajectory. We then systematically classify existing and emerging PCSK9‐targeting agents, including monoclonal antibodies, oral inhibitors, and epigenetic editing therapies. Furthermore, we consolidate preclinical and clinical findings supporting their roles in neuroprotection, vasculoprotection, and improved functional outcomes after stroke. Finally, we discuss future directions involving personalized treatment strategies and combinatorial regimens, aiming to pave the way for a new era of precision medicine in neurovascular care.

## 2 PCSK9 Biology and Physiology

**FIGURE 1:**
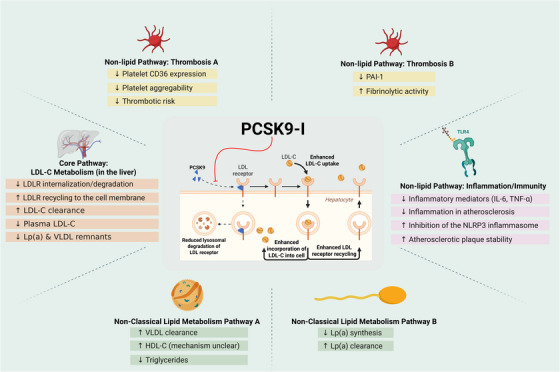
Summary of PCSK9 inhibitor mechanisms. PCSK9 inhibitors (PCSK9‐i) primarily lower LDL‐cholesterol (LDL‐C) by blocking PCSK9‐mediated degradation of hepatic LDL receptors (LDLR), enhancing LDL clearance. Beyond LDLR‐dependent effects, PCSK9‐i exert multiple benefits via LDLR‐independent pathways. These include anti‐inflammatory effects (suppressing TLR4/NF‐κB, NLRP3 inflammasome, and cytokine release), antioxidant actions (reducing NOX‐derived ROS and enhancing Nrf2/HO‐1), and improved endothelial function (via eNOS/NO upregulation and reduced adhesion molecules). Additionally, PCSK9‐i may modulate immune responses (reducing macrophage CD36 and T‐cell activation), thrombosis (lowering platelet activity and tissue factor), and metabolic pathways (potentially improving insulin sensitivity via SIRT1/AMPK). They also influence plaque stability by decreasing smooth muscle cell proliferation and oxidative stress.

PCSK9, a hepatocyte‐derived serine protease, regulates LDL‐C by binding the LDL receptor (LDLR), redirecting it to lysosomal degradation instead of recycling, thereby reducing hepatic LDL‐C clearance. Gain‐of‐function mutations elevate cardiovascular risk, while LOF variants lower LDL‐C and atherosclerosis susceptibility. Mechanistically, PCSK9 disrupts sorting nexin 17 (SNX17)‐mediated LDLR recycling in endosomes, and mutations impairing this pathway confer resistance to PCSK9‐i. Beyond lipid metabolism, PCSK9 promotes inflammation (e.g., NLRP3 activation via lipid accumulation), thrombosis (e.g., platelet activation via CD36), and plaque vulnerability. PCSK9‐i (e.g., evolocumab, alirocumab) neutralize circulating PCSK9, restoring LDLR recycling and reducing LDL‐C by 50–60%, while pleiotropic effects (e.g., plaque stabilization, anti‐inflammation) contribute to cardiovascular risk reduction independently of LDL‐C lowering (Figure 1).

### 2.1 Biosynthesis and Structural Basis of Function

**FIGURE 2:**
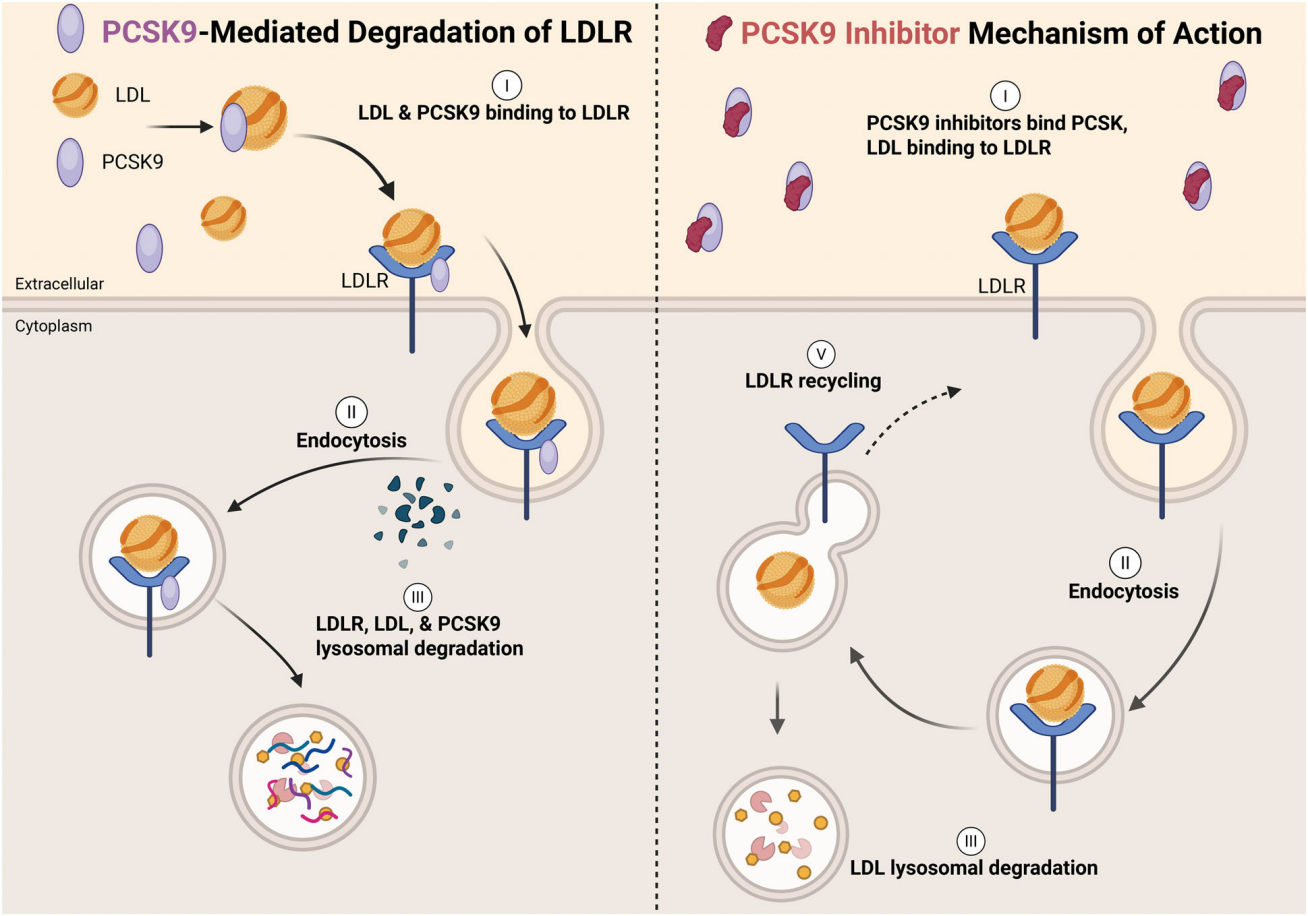
Primary biological function of PCSK9 inhibitors. PCSK9 inhibitors (PCSK9‐i) primarily function by regulating LDL cholesterol (LDL‐C) metabolism through their interaction with the LDL receptor (LDLR). By binding to and inhibiting circulating PCSK9, these therapeutics prevent PCSK9‐mediated degradation of LDLR in hepatocytes. This stabilization of LDLR enhances hepatic clearance of LDL‐C from plasma, leading to significant reductions in circulating LDL‐C levels. Beyond this central mechanism, PCSK9‐i exhibit secondary effects on lipid metabolism, including modest reductions in lipoprotein(a) [Lp(a)] and very‐low‐density lipoprotein (VLDL) remnants. While their primary action is LDLR dependent, emerging evidence suggests potential LDLR‐independent effects on inflammatory pathways, endothelial function, and platelet activity.

PCSK9, a hepatocyte‐derived serine protease belonging to the proprotein convertase family, plays a pivotal role in modulating protein homeostasis through proteolytic processing and degradation of secreted proteins [11, 12]. Its primary biological function centers on regulating LDL cholesterol (LDL‐C) metabolism via interaction with the LDLR (Figure 2). Mechanistically, PCSK9 binds to LDLR on hepatocyte membranes, redirecting the receptor toward lysosomal degradation rather than cellular recycling, thereby limiting hepatic LDL‐C clearance [13, 14]. This intervention reduces hepatic LDLR density by 50–70%, diminishes LDL‐C clearance capacity by 30–40%, and elevates circulating LDL‐C levels [15, 16, 17, 18].

Gain‐of‐function mutations (e.g., D374Y, S127R) amplify PCSK9 activity, causing autosomal dominant hypercholesterolemia (LDL‐C > 190 mg/dL) and accelerating atherosclerosis progression. Conversely, LOF variants (e.g., R46L, Y142X) reduce circulating PCSK9 by 40%, lower LDL‐C by 15–28%, and decrease cardiovascular risk by 47% [19, 20, 21]. These findings not only validate PCSK9 as a critical therapeutic target but also highlight its potential for personalized interventions in patients with heightened sensitivity to LDL‐C atherogenicity [22]. PCSK9‐i constitute a significant therapeutic advancement in lipid management, primarily targeting the LDLR degradation pathway [23]. Consequently, managing these patients necessitates genetic diagnosis independent of LDLR function assessment and consideration of alternative therapeutic agents. By neutralizing circulating PCSK9 via monoclonal antibody administration (e.g., evolocumab, alirocumab), PCSK9‐i disrupt the PCSK9–LDLR interaction, thereby preserving surface LDLR density on hepatocyte membranes. This augmentation of LDLR availability significantly increases the hepatic clearance rate of LDL particles, resulting in robust plasma LDL‐C reduction of 50–60%. This efficacy profile has been conclusively demonstrated in pivotal cardiovascular outcomes trials, including FOURIER (in patients with atherosclerotic cardiovascular disease [ASCVD]) and ODYSSEY OUTCOMES (in high‐risk cardiovascular cohorts) [24, 25, 26, 27, 28].

It is widely held within the lipidology community that the primary cardiovascular benefits of PCSK9 inhibition derive significantly from robust reductions in LDL‐C. Proteomic analyses, exemplified by the Systematic Protein Investigative Research Environment (SPIRE) trial, demonstrate that the modulatory effects of PCSK9‐i on circulating inflammatory markers are wholly concordant with the magnitude of achieved LDL‐C lowering. Crucially, no distinct inflammatory signaling pathways independent of LDL‐C reduction have been identified. Genetic studies of naturally occurring PCSK9 LOF variants provide robust corroborative evidence, strongly supporting LDL‐C reduction as the principal mediator of cardiovascular protection.

However, while the canonical mechanism of action (MoA) of PCSK9‐i involves enhanced recycling and stabilization of the LDLR, emerging mechanistic investigations suggest potential contributions from additional, noncanonical pathways that may operate independently of LDL‐C reduction. From a clinical perspective, the paramount therapeutic objective remains the attainment of guideline‐recommended LDL‐C targets. Any potential ancillary benefits arising from mechanistic nuances are likely to constitute secondary considerations, potentially offering context‐specific advantages beyond LDL‐C lowering per se [29].

### 2.2 Canonical Role in LDLR Recycling and Cholesterol Homeostasis

#### 2.2.1 Compartmentalized LDLR Trafficking: SNX17‐Dependent Recycling Pathway

Research elucidating the SNX17‐dependent LDLR recycling pathway has established that PCSK9 binding prevents LDLR acidification‐induced conformational changes within endosomal compartments. This impairment disrupts critical binding interactions between SNX17 and the evolutionarily conserved “NPxY” endocytic sorting motif in the LDLR cytosolic domain. Consequently, LDLR trafficking to recycling endosomes is compromised, resulting in receptor retention within endolysosomal compartments and proteolytic degradation by lysosome‐resident acid hydrolases.

Notably, FH patients harboring *LDLR* LOF variants impacting intracellular trafficking pathways exhibit diminished therapeutic responsiveness to PCSK9‐i. This pharmacoresistance necessitates comprehensive genetic characterization independent of standard LDLR functional assays and mandates consideration of alternative lipid‐lowering strategies. These clinical observations substantiate the fundamental role of compartmentalized LDLR trafficking regulation in cholesterol homeostasis.

Crucially, experimental SNX17 ablation in macrophages completely abrogates PCSK9‐mediated LDLR degradation, demonstrating that localized receptor recycling operates independently of systemic LDL‐cholesterol (LDL‐C) clearance pathways. This mechanistic distinction provides the molecular foundation whereby PCSK9 inhibition enhances macrophage uptake and degradation of oxidized LDL (oxLDL) particles through LDLR pathway modulation—even when plasma LDL‐C levels remain unaltered. The pathway demonstrates tissue‐specific regulation of lipoprotein metabolism beyond hepatic clearance mechanisms [30, 31].

#### 2.2.2 Plaque‐Specific Macrophage Reprogramming: LDLR‐Independent Modulation of Atherogenic Mechanisms

Transplantation of bone marrow overexpressing human PCSK9 (hPCSK9) into apolipoprotein E‐deficient (apoE^−^/^−^) mice—which retain functional LDLR expression—demonstrated specific hPCSK9 accumulation within atherosclerotic plaques. This localized overexpression induced significant downregulation of both LDLR and LDLR‐related protein 1 (LRP1) on macrophage surfaces, markedly compromising oxLDL clearance capacity [32, 33]. Administration of PCSK9‐i reversed this phenotype, restoring macrophage phagocytic function and reducing foam cell formation. Notably, parallel experiments in LDLR‐deficient (LDLR^−^/^−^) recipients revealed that circulating PCSK9 propagates macrophage activation and accelerates venous graft lesion progression through LDLR‐independent pathways.

These observations indicate that targeted LDLR upregulation within plaque‐resident macrophages improves intraplaque lipid homeostasis independently of systemic lipid parameters. Critically, PCSK9‐i achieve three synergistic antiatherogenic effects at the plaque level: specific upregulation of macrophage LDLR expression; enhanced oxLDL efflux and catabolism; attenuation of NLRP3 inflammasome activation. Importantly, these localized beneficial effects persist even in contexts of suboptimal systemic LDL‐C concentrations [34, 35]. This suggests that modulating macrophage LDLR expression within atherosclerotic lesions confers therapeutic advantages without necessitating further reduction of circulating LDL‐C levels, highlighting a distinct dimension of PCSK9 inhibition beyond conventional lipid management.

#### 2.2.3 Anti‐Inflammatory Effects Beyond LDL‐C Reduction: Pleiotropic Plaque Stabilization

In murine models with systemically controlled LDL‐C concentrations, *local* PCSK9 inhibitor administration decreased inflammatory monocyte infiltration into atherosclerotic plaques by 32% while simultaneously enhancing fibrous cap stability and collagen content [36]. These findings demonstrate that the anti‐inflammatory and plaque‐stabilizing properties of PCSK9 inhibition operate independently of circulating lipoprotein levels, establishing a molecular foundation for their pleiotropic vasculoprotective actions beyond conventional lipid‐lowering benefits. Concurrently, *Gynostemma pentaphyllum*‐derived total saponins modulate atherosclerotic pathophysiology through: suppression of hepatic PCSK9 transcription; activation of macrophage autophagy‐lysosomal degradation pathways (lipophagy); enhanced LDLR recycling via SNX17 upregulation; reduction of intracellular cholesteryl ester accumulation; inhibition of NLRP3 inflammasome‐mediated cytokine secretion [37, 38, 39, 40].

Collectively, these mechanistic insights substantiate the non‐lipid‐lowering therapeutic benefits of PCSK9‐i—particularly in patients achieving very low LDL‐C levels (<55 mg/dL). This has distinct clinical relevance for: FH patients exhibiting pharmacoresistance to conventional therapies; individuals with high‐risk plaques demonstrating active inflammatory signatures.

### 2.3 Noncanonical (Pleiotropic) Functions in Inflammation, Thrombosis, and Immunity

#### 2.3.1 LDLR‐Dependent Inflammasome Priming via Intracellular Lipid Accumulation

PCSK9 significantly enhances mRNA expression of proinflammatory cytokines tumor necrosis factor (TNF)‐α and interleukin (IL)‐1β (increased by 40–45%) while concurrently elevating anti‐inflammatory mediators IL‐10 and Arg1 (30–44% increase) in lipopolysaccharide‐stimulated macrophages. This bidirectional immunomodulatory effect demonstrates strict LDLR dependence, as evidenced by complete abrogation in LDLR^−^/^−^ macrophages.

Through LDLR downregulation, PCSK9 impairs macrophage clearance of oxLDL, resulting in intracellular cholesterol crystallization. This lipid accumulation triggers NLRP3 inflammasome assembly and caspase‐1 activation, establishing a direct pathophysiological link between impaired cholesterol efflux and sterile inflammation.

Pharmacological PCSK9 inhibition attenuates this inflammatory cascade by restoring macrophage LDLR surface expression, thereby enhancing cholesterol efflux capacity and suppressing NLRP3 inflammasome activity independently of systemic lipid parameters [41, 42].

#### 2.3.2 Pathophysiological Integration of PCSK9 in Atherosclerotic Plaques

PCSK9 demonstrates significant extrahepatic localization within arterial wall microenvironments, where it exerts pronounced proatherogenic influence. These local effects critically drive plaque vulnerability through distinct pathomorphological mechanisms that compromise structural integrity and compositional stability of advanced atheromata.

Beyond its canonical regulation of hepatic LDLR recycling and systemic lipid homeostasis, PCSK9 participates in diverse vascular pathophysiological cascades: Amplification of proinflammatory signaling cascades; modulation of VSMC phenotypic switching; promotion of VSMC proliferative and migratory phenotypes; enhancement of thrombogenicity through platelet reactivity and coagulation cascade modulation.

#### 2.3.3 Proinflammatory Mechanisms of PCSK9 in Atherogenesis

Epidemiological analyses have revealed a significant positive correlation between circulating PCSK9 levels and systemic inflammatory biomarkers, including high‐sensitivity C‐reactive protein (hs‐CRP), underscoring its role in modulating inflammatory responses [11, 43, 44]. Importantly, clinical studies of PCSK9‐i demonstrate their capacity to remodel the inflammatory microenvironment within atherosclerotic plaques: immunohistochemical analyses show marked reductions in proinflammatory proteins (NLRP3, IL‐1β, TNF‐α) alongside increased collagen deposition, indicating enhanced plaque stability [45, 46]. The mechanistic link between PCSK9 activity and inflammatory biomarker elevation provides a compelling rationale for targeting this pathway in patients with high cardiovascular risk, particularly those exhibiting elevated hs‐CRP or unstable plaque morphology [45, 47]. Serial coronary computed tomography angiography studies demonstrate that combined administration of PCSK9‐i and statins attenuates total atheroma volume progression by 6–9% while enhancing fibrotic tissue content by 12–15%. These structural modifications correspond to a 27% reduction in recurrent major adverse cardiovascular events (MACEs) [48, 49, 50, 51].

Moreover, experimental studies in cholesteryl ester transfer protein‐expressing murine models reveal dramatic attenuation of plaque progression: 40–50% reduction in aortic arch lipid deposition; 35–45% decrease in systemic inflammatory biomarkers (IL‐6, TNF‐α); 25–30% increase in fibrous cap thickness; 40% reduction in necrotic core cross‐sectional area [52, 53].

#### 2.3.4 Structural‐Driven VSMC Apoptosis: Necrotic Core Expansion via TLR4/Mitochondrial Axis

Structural studies demonstrate that the C‐terminal cysteine‐rich domain of PCSK9 binds to TLR4, activating the downstream NF‐κB signaling pathway, which drives the secretion of proinflammatory cytokines (e.g., IL‐6, TNF‐α) and promotes aberrant VSMC migration [45]. These mechanisms enable PCSK9 to regulate VSMC differentiation, proliferation, and motility, thereby accelerating atherosclerotic plaque development [52, 54]. Notably, PCSK9 induces mitochondrial dysfunction and apoptosis in VSMCs through morphology‐dependent pathways, processes strongly associated with increased vulnerability of carotid plaques [55, 56]. Cohort studies demonstrate a proportional relationship between serum PCSK9 levels and necrotic core volume within plaques (*r* = 0.58, *p* < 0.001), suggesting a direct mechanistic link to plaque destabilization [57]. The landmark STAINLAS trial further substantiates this association, revealing that elevated PCSK9 levels independently contribute to carotid plaque formation (OR 2.3, 95% confidence interval [CI] 1.6–3.4), even after adjusting for traditional risk factors [58]. These pathomorphological features develop independently of arterial lumen stenosis, emphasizing the critical role of PCSK9 in driving plaque vulnerability through inflammation‐mediated mechanisms [59].

#### 2.3.5 Endothelial Salvage Pathways: Sirtuin‐Mediated Metabolic Revitalization

Vascular endothelial injury initiates a cascade of pathophysiological events: platelet adhesion, blood–brain barrier (BBB) compromise, and vasospasm—collectively promoting thrombogenesis and cerebral ischemia [60]. Clinical evidence demonstrates that PCSK9‐i preserve endothelial integrity through multimodal mechanisms: *coronary vasculature*: restoration of endothelial nitric oxide synthase (eNOS) activity enhances flow‐mediated dilation (35% improvement versus baseline) [61]; *diabetic vasculopathy*: combination therapy with glucose‐lowering agents augments endothelial progenitor cell (EPC) functionality (28% increase in EPC activity versus monotherapy) in type 2 diabetes patients [62, 63]. The landmark FOURIER trial established evolocumab‐mediated reduction in ischemic stroke incidence, attributable to both intensive LDL‐C reduction and documented suppression of platelet aggregation [64, 65, 66].

Preclinical studies indicate PCSK9‐i mitigate mitochondrial oxidative stress and improve vascular function via Sirtuin pathway activation (particularly SIRT3) in endothelial cells, mechanistically involving: upregulation of mitochondrial superoxide dismutase; enhanced endothelial metabolic flexibility [36, 67, 68, 69]. Additionally, immunomodulatory actions—including inhibition of oxLDL‐induced dendritic cell maturation—have been experimentally demonstrated, though their clinical translatability requires further validation.

#### 2.3.6 Prothrombotic Circuitry: CD36‐Mediated Platelet Priming and Coagulation Cascade

Thrombosis, defined as the formation of a blood clot (thrombus) at sites of vascular injury or endothelial disruption, represents a critical pathological process underlying ischemic heart disease, ischemic stroke, and venous thromboembolism (VTE) [70, 71]. Beyond its role in atherosclerosis, PCSK9 enhances platelet reactivity by upregulating lipid‐mediated signaling pathways, thereby increasing blood hypercoagulability and thrombosis susceptibility [72]. Specifically, PCSK9 induces tissue factor expression in monocytes and elevates coagulation factor VIII (FVIII) levels, directly activating the extrinsic coagulation cascade [73]. Furthermore, PCSK9 interacts with the platelet CD36 receptor, amplifying platelet activation through lipid raft reorganization and prothrombotic signaling [72, 74, 75]. This interaction is functionally significant, as demonstrated by studies showing that CD36 knockdown or PCSK9‐i (e.g., evolocumab) substantially reduce platelet aggregation and thrombus formation in vivo [76].

Therapeutic interventions using PCSK9‐i counteract these effects through dual mechanisms: (1) reducing LDL‐C to mitigate lipid‐driven platelet hyperreactivity, and (2) blocking PCSK9–CD36 interactions to suppress direct platelet activation [76]. Additionally, PCSK9‐i moderately lower lipoprotein(a) [Lp(a)] levels, further attenuating thrombotic risk through multiply mechanisms [77].

PCSK9‐i exert atheroprotective effects through multifaceted mechanisms: Beyond their primary action of significantly reducing circulating LDL‐C levels via antagonism of PCSK9‐mediated LDLR catabolism, these agents target local arterial PCSK9 activity. They suppress proinflammatory signaling within the vascular wall, thereby attenuating PCSK9's adverse contributions to plaque composition—including enhanced macrophage infiltration and compromised fibrous cap integrityand modulating plaque vulnerability indices. Crucially, emerging evidence indicates direct inhibition of VSMC proliferation and migration, alongside potential interference with prothrombotic pathways. Consequently, PCSK9‐i synergistically ameliorate lipid profiles, promote plaque stabilization, and retard atherosclerosis progression through both lipid‐dependent and lipid‐independent mechanisms. This multiphasic activity translates to reduced incidence of MACEs [78].

## 3 Therapeutic Targeting of PCSK9: From Mechanisms to Agents

PCSK9‐i comprise two primary classes: monoclonal antibodies (e.g., evolocumab, alirocumab) and small‐interfering RNA (siRNA) therapeutics (e.g., inclisiran). Both enhance hepatic LDLR recycling, reducing LDL‐C by 50–60% and mitigating cardiovascular risk via pleiotropic pathways. Clinical trials (e.g., FOURIER, FOURIER‐OLE) demonstrate that evolocumab sustains LDL‐C suppression (<20 mg/dL), lowering cardiovascular mortality by 15% and ischemic stroke risk by 21% over 8.4 years. Imaging studies (GLAGOV, HUYGENS) further link PCSK9 inhibition to atherosclerotic plaque stabilization and regression. Inclisiran's biannual dosing enables prolonged LDL‐C control but is less optimal for rapid reduction. Synergy with statins achieves additive LDL‐C lowering (60–70%) and reduces VTE risk by 34%. Emerging CRISPR‐based epigenetic editing promises durable LDL‐C management, while existing classes support aggressive LDL‐C targets (<55 mg/dL) in high‐risk ASCVD patients.

### 3.1 Pharmacological Modalities: Monoclonal Antibodies, siRNA, and Emerging Platforms

**FIGURE 3:**
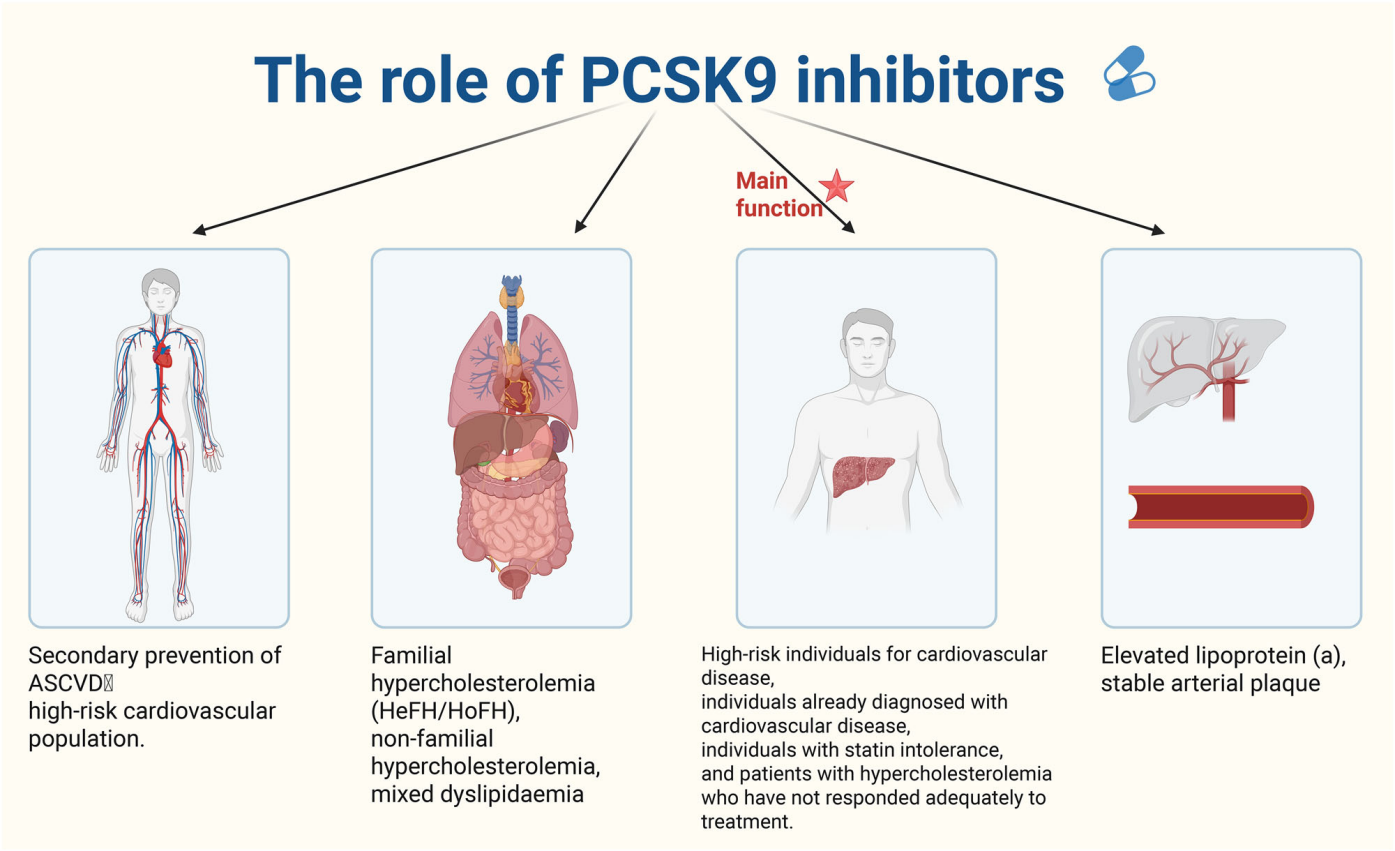
The role of PCSK9 inhibitors. PCSK9 inhibitors (PCSK9‐i) are a revolutionary class of therapeutics that primarily function by significantly lowering low‐density lipoprotein cholesterol (LDL‐C) levels through inhibition of PCSK9‐mediated degradation of hepatic LDL receptors, resulting in enhanced LDL particle clearance from circulation. Beyond their potent lipid‐lowering effects (achieving 50–60% reductions in LDL‐C), these agents provide robust cardiovascular protection by slowing atherosclerotic plaque progression, stabilizing vulnerable plaques, and reducing the incidence of MACEs in high‐risk patients. Additionally, PCSK9‐i exhibit multiple vascular benefits, including endothelial stabilization through improved nitric oxide bioavailability, reduced expression of adhesion molecules (VCAM‐1/ICAM‐1), and decreased endothelial inflammation. They also demonstrate anti‐inflammatory properties by modulating the NLRP3 inflammasome and suppressing proinflammatory cytokines, as well as antioxidant effects via upregulation of Nrf2/HO‐1 pathways.

PCSK9‐i comprise two principal classes: monoclonal antibodies (e.g., evolocumab, alirocumab) and siRNA therapeutics (e.g., inclisiran) (Table 1) [79, 80]. Both facilitate hepatic LDLR recycling, leading to a median reduction of 50–60% in circulating LDL‐C levels [81, 82, 83]. Beyond lipid lowering, these agents mitigate cardiovascular risk through pleiotropic pathways, solidifying their role as foundational therapies in contemporary cardiovascular disease management (Figure 3) [84, 85].

**TABLE 1:** Globally marketed PCSK9 inhibitors.

Drug name	NCT/ID	Indications	Global top stage
Ongericimab	05325203	Approved (3): primary hypercholesterolemia; dyslipidemia; hyperlipidemia Investigational (7): heterozygous familial hypercholesterolemia; mixed hyperlipidemia; pure familial hypercholesterolemia; nonfamilial hypercholesterolemia Clinical stage III: hypercholesterolemia Clinical stage II: cardiovascular disease Clinical application: tumors	Approved for marketing (2024‐10‐09)
Undisclosed, AFFiRiS AG (PCSK9, hypercholesterolemia, biepitopic)	02508896	In progress: (preclinical) hypercholesterolemia	Preclinical (2024‐12‐13)
SAL‐003	None	In research (4): Clinical stage III: mixed hyperlipidemia; hypercholesterolemia Clinical stage I: hyperlipidemia Clinical application: dyslipidemia	Clinical phase III (2023‐07‐13)
Evolocumab	02304484	In progress (11): Registry application: cardiovascular events Clinical phase III: type 2 diabetes mellitus; HIV infection; ST‐segment elevation myocardial infarction; coronary artery disease; arteritis; acute coronary syndrome; heterozygous familial hypercholesterolemia; hyperlipidemia Clinical stage II: metastatic desmoplasia‐sensitive prostate cancer Clinical phase I: myocardial infarction Approved (10): atherosclerosis; atherosclerotic cardiovascular disease; primary hypercholesterolemia; familial hypercholesterolemia; heart disease; cardiovascular disease; mixed hyperlipidemia; heterozygous familial hypercholesterolemia; dyslipidemia; hypercholesterolemia	Approval to market (2015‐07‐17)
B‐1655	02613871	In research (2): clinical phase I: hypercholesterolemia; hyperlipidemia	Clinical phase I (2021‐01‐26)
Recombinant human anti‐PCSK9 monoclonal antibody	2000031373	In progress: (clinical phase I) hypercholesterolemia	Clinical phase I (2020‐05‐06)
ANTI PCSK9 MAB	03355027	In progress: (clinical phase I) hypercholesterolemia	Clinical phase I (2018‐04‐13)
DS‐9001	None	Termination: dyslipidemia	Clinical phase I (2017‐05‐11)
Undisclosed, Aanastra Inc (PCSK9, familial hypercholestrolemia, peptide–mRNA)	None	In progress: (preclinical) hypercholesterolemia	Preclinical
PBGENE–PCSK9	None	In progress: (preclinical) familial hypercholesterolemia	Preclinical
SGPCSK9i	None	In progress: (preclinical) hypercholesterolemia	Preclinical

*Data sources*: https://vip.yaozh.com/home.

Cardiovascular outcome trials, such as FOURIER and its open‐label extension FOURIER‐OLE, demonstrated that evolocumab achieves sustained LDL‐C reductions (median <20 mg/dL), alongside a 15% reduction in cardiovascular mortality (HR 0.85) and a 21% lower risk of ischemic stroke (HR 0.79) over a median follow‐up of 8.4 years [86, 87, 88]. Imaging studies including GLAGOV and HUYGENS further indicated that evolocumab promotes atherosclerotic plaque stabilization and regression by increasing fibrous cap thickness and reducing lipid core volume [89, 90].

Inclisiran, an siRNA therapy, targets and degrades PCSK9 mRNA within hepatocytes, enabling biannual dosing and prolonged LDL‐C reduction [91, 92, 93]. While both therapeutic classes achieve substantial LDL‐lowering, monoclonal antibodies are often preferred in scenarios requiring rapid LDL‐C reduction, such as in high‐risk patients with established ASCVD [94, 95, 96].

When combined with statins, PCSK9‐i exhibit synergistic effects. For instance, alirocumab added to high‐intensity statin therapy reduces LDL‐C by an additional 60–70% compared with statin monotherapy [97, 98, 99]. Meta‐analyses of randomized trials also indicate that this combination confers a 34% reduction in the risk of VTE (RR 0.66, 95% CI 0.49–0.89) [100, 101, 102].

Emerging strategies, such as CRISPR‐based epigenetic editing of PCSK9, hold potential for durable LDL‐C management but remain under investigation [103]. Despite differing mechanisms—extracellular neutralization for monoclonal antibodies versus intracellular gene silencing for siRNA therapies—both classes effectively achieve significant LDL‐C reduction, supporting a treatment paradigm aiming for LDL‐C levels below 55 mg/dL in high‐risk ASCVD patients [104, 105].

### 3.2 MoA: Neutralizing PCSK9 and Restoring LDLR Function

PCSK9‐i effectively ameliorate dyslipidemia by selectively binding to PCSK9, thereby preventing its interaction with the LDLR. This inhibition increases LDLR availability on hepatocytes, enhancing LDL‐C clearance and significantly reducing circulating LDL‐C levels by 50–60%, as demonstrated in clinical trials [106, 107, 108, 109].

The landmark FOURIER trial showed that PCSK9‐i not only achieve profound LDL‐C reduction (median LDL‐C <25 mg/dL) but also confer robust cardiovascular benefits, including a 15% reduction in cardiovascular mortality and a 21% lower risk of ischemic stroke. These benefits were sustained over long‐term follow‐up [86, 87].

In high‐risk patients with recent acute coronary syndromes (ACSs) and baseline LDL‐C near 70 mg/dL despite optimized statin therapy, PCSK9 inhibition provides additional clinical benefit when Lp(a) concentrations exceed 30 mg/dL, reducing MACEs by 23% [110, 111].

Thus, PCSK9‐i have emerged as a pivotal therapeutic option for lipid management, particularly in patients who fail to achieve adequate LDL‐C lowering with maximally tolerated statins and ezetimibe [112, 113, 114, 115]. Beyond lipid‐lowering, these agents may also improve stroke prognosis through pleiotropic mechanisms, such as plaque stabilization, endothelial function restoration, and attenuation of thrombotic risk [84, 85].

## 4 Preclinical Evidence of PCSK9 Inhibition Beyond Lipid‐Lowering

Preclinical studies have been instrumental in elucidating the multifaceted mechanisms by which PCSK9 inhibition confers protection beyond lipid lowering. The evidence can be categorized into four primary, interlinked pathways: direct neuroprotection, stabilization of the vascular compartment, and systemic anti‐inflammatory and immunomodulatory effects.

### 4.1 Antiatherosclerotic and Vasoprotective Effects

Preclinical studies in APOE*3Leiden.CETP transgenic mice, a well‐established model of atherosclerosis, revealed that PCSK9 gene knockdown exerts potent anti‐inflammatory effects. Genetic inhibition of PCSK9 significantly reduced TLR4 and NF‐κB expression in microglia (*p* < 0.01), concurrently lowering intraplaque levels of proinflammatory mediators such as IL‐1β and MCP‐1 (*p* < 0.05). This attenuation of vascular inflammation was mediated through dual mechanisms: suppression of the TLR4/NF‐κB signaling pathway and reduced uptake of oxLDL by macrophages. The study further demonstrated that PCSK9 inhibition decreases oxidative stress markers and enhances plaque stability by increasing collagen content, underscoring its therapeutic potential for mitigating inflammation‐driven atherosclerotic progression [116].

In a mouse model of abdominal aortic aneurysm (AAA), genetic knockout (Pcsk9^−^/^−^) or pharmacological inhibition (PCSK9‐targeting antibodies/siRNA) significantly attenuated vascular inflammation and structural degeneration. PCSK9 deficiency reduced aortic macrophage infiltration by 60–70% (*p* < 0.01) and elastin degradation by 40–50%, while simultaneously suppressing matrix metalloproteinase‐9 (MMP‐9) expression and enhancing the SIRT1/NF‐κB pathway, which preserved vascular integrity. These findings provide a robust preclinical foundation for translating PCSK9‐targeted therapies into clinical strategies for AAA management, particularly in patients with inflammatory‐driven vascular remodeling [36, 117].

Compared with prior research, this study more clearly delineates the crucial role of PCSK9 in plaque stabilization and extracellular matrix remodeling. Importantly, activation of the SIRT1/NF‐κB pathway upon PCSK9 inhibition suggests epigenetic regulation of inflammation, offering a deeper mechanistic basis for PCSK9‐targeted therapies. However, current evidence remains limited to preclinical models, and clinical translation necessitates validation of these mechanisms in human vascular tissues. Furthermore, exploring synergies between PCSK9‐i and other anti‐inflammatory agents (e.g., IL‐1β inhibitors) could help maximize the benefits of multipathway combination treatments.

### 4.2 Neuroprotective and BBB Stabilizing Effects

In atherosclerosis‐prone mouse models, the combination of alirocumab (a PCSK9 monoclonal antibody) with statins demonstrated neuroprotective potential by stabilizing atherosclerotic plaques and mitigating vascular endothelial damage through inhibition of inflammatory cytokines such as IL‐6 and TNF‐α, thereby indirectly preserving neurological function [118]. Similarly, in FH models, PCSK9 monoclonal antibodies exhibited neuroprotective effects by modulating the immune microenvironment, reducing neuroinflammation, and enhancing blood–brain barrier integrity [119].

In a mouse model of Alzheimer's disease, the PCSK9 inhibitor evolocumab demonstrated neuroprotective effects by enhancing BBB integrity. Mechanistically, evolocumab reduced MMP‐9 secretion through inhibition of microglial activation, thereby attenuating amyloid‐β (Aβ) deposition‐induced BBB disruption. These findings highlight a novel therapeutic avenue for neurodegenerative disorders, extending PCSK9‐i’ benefits beyond lipid modulation to direct neurovascular protection [120].

In murine models, evolocumab significantly reduces plasma LDL‐C without affecting cerebral cholesterol levels—a contrast to statins like atorvastatin, which penetrate the BBB and suppress brain cholesterol synthesis [121]. In vitro BBB models reveal that PCSK9‐i maintain barrier function by enhancing LRP1‐mediated Aβ clearance, independent of central cholesterol metabolism [122]. oxLDL‐induced endothelial damage via LOX‐1 receptor activation is mitigated by PCSK9‐i through dual pathways: reduction of oxLDL levels inhibits endothelial apoptosis and BBB disruption and attenuation of aortic inflammation in AAA models by suppressing macrophage infiltration and MMP‐9 activity, concurrently reducing serum TNF‐α and IL‐6 (*p* < 0.01) while upregulating tight junction proteins (e.g., claudin‐5) to fortify BBB integrity [36].

Elevated homocysteine levels, associated with plaque instability, are counteracted by PCSK9‐i through slowing intraplaque neovascularization and inflammation, indirectly stabilizing BBB integrity, and lowering oxLDL levels and inhibiting microglial activation to reduce neuroinflammation and endothelial dysfunction. These interconnected mechanisms form a comprehensive BBB protective network, positioning PCSK9‐i as unique therapeutic agents capable of addressing both vascular and neurological aspects of cerebrovascular disease [123].

Further insights emerged from studies using hyperlipidemic mice subjected to ischemic stroke via middle cerebral artery occlusion (MCAO). This intervention reduced infarct volume by 35–40% and improved functional recovery, underscoring the therapeutic potential of PCSK9 inhibition in stroke management [124]. Complementary research in a transient MCAO model revealed that PCSK9 inhibitor treatment upregulated glycoprotein nonmetastatic melanoma protein B, a neuroprotective factor, resulting in a 30% reduction in infarct volume and enhanced neuronal survival. This suggests that PCSK9‐i may amplify endogenous neuroprotective pathways, offering dual benefits in ischemia–reperfusion injury [125].

PCSK9‐i provide neuroprotection mainly by stabilizing the BBB and modulating immune responses. These actions support emerging strategies focused on cerebrovascular integrity. For instance, similar to sigma‐1 receptor agonists such as DMT, PCSK9‐i help reduce neuroinflammation and maintain barrier function by stabilizing tight junction proteins and inhibiting release of proinflammatory cytokines. Key pathways involved include CX43–PARP1–NAD⁺ and PI3K/AKT, which are crucial for BBB maintenance under disease conditions. By mitigating IL‐6 and TNF‐α‐mediated inflammation, PCSK9‐i highlight potential immunometabolic therapies for stroke and neurodegeneration. However, their exact mechanisms in neuroinflammatory cascades need further study. Future research should investigate whether PCSK9 regulation directly influences astrocyte activation or endothelial permeability. Additionally, since monoclonal antibodies poorly cross the intact BBB, clinical applications may require combination with permeabilization techniques like focused ultrasound or nanoparticle delivery to improve efficacy.

### 4.3 Antithrombotic Effects

In a venous thrombosis model induced by inferior vena cava ligation, PCSK9‐deficient mice exhibited a 40–50% reduction in thrombus size compared with wild‐type controls, accompanied by significantly lower serum soluble P‐selectin (sP‐selectin) levels (↓35%, *p* < 0.01). This suggests PCSK9 modulates thrombogenesis by enhancing platelet α‐granule release and endothelial activation [126]. Further mechanistic studies revealed that PCSK9 regulates thrombus formation via interactions with platelet membrane receptors (e.g., CD36) and endothelial adhesion molecules (e.g., P‐selectin), promoting leukocyte–platelet aggregate formation [127, 128]. Treatment with evolocumab, a PCSK9 inhibitor, attenuated these prothrombotic effects by 60–70%, normalizing platelet reactivity. CD36 knockout models demonstrated that PCSK9's thrombogenic actions are CD36‐dependent, mediated through activation of the Src kinase/MAPK signaling axis (ERK5, JNK) and downstream pathways, such as ROS production, p38MAPK/cytosolic phospholipase A2 activation and cyclooxygenase‐1 (COX‐1)‐dependent thromboxane A2 (TXA2) synthesis. Notably, aspirin administration completely abolished PCSK9‐enhanced platelet activation and thrombosis in vivo, suggesting synergistic therapeutic potential when combining PCSK9‐i with antiplatelet agents.

PCSK9 deficiency in mice confers strong protection against thrombosis, underscoring its critical role in platelet‐driven thrombotic processes and supporting therapeutic strategies targeting the thrombotic microenvironment. Aspirin completely reverses PCSK9‐enhanced thrombosis, confirming the central involvement of the COX‐1/TXA2 pathway and highlighting the value of combination therapies. Unlike conventional antiplatelet agents (e.g., aspirin, clopidogrel), which increase bleeding risk, PCSK9 inhibition may allow more precise targeting of pathological thrombosis without compromising hemostasis. Future work should explore synergies between PCSK9‐i and emerging antithrombotic agents (e.g., hirudin derivatives or natural anticoagulant peptides) to enhance efficacy while limiting bleeding risks. Further investigation is also needed into the interplay between PCSK9, CD36, and oxidative stress pathways to uncover upstream regulatory mechanisms and identify biomarkers for patient stratification.

### 4.4 Immunomodulatory Effects

In vitro studies using macrophage and endothelial cell models demonstrate that PCSK9 in VSMCs is regulated through the TLR4–SAPK/JNK signaling pathway, a critical mediator of inflammatory and metabolic processes. VSMC‐derived PCSK9 reduces monocyte LDLR expression and impairs LDL‐C/LDLR‐mediated CCR2 expression on monocytes, a mechanism essential for monocyte recruitment and atherogenesis [47, 129]. This inhibition also elevates MHC class I molecule expression on tumor cells within the tumor microenvironment, facilitating CD8+ T cell recognition and tumor cell elimination [130, 131, 132].

In a pristane‐induced systemic lupus erythematosus (SLE) mouse model, pharmacological inhibition of PCSK9 significantly attenuated systemic inflammation, as evidenced by a 40–50% reduction in proinflammatory cytokines (IL‐17, TNF‐α) and increase in anti‐inflammatory TGF‐β levels (*p* < 0.01). Clinical validation in SLE patients revealed a strong positive correlation between circulating PCSK9 levels and CRP (*p* < 0.001), further implicating PCSK9 in systemic inflammatory cascades. These findings position PCSK9 as a novel therapeutic target for autoimmune disorders, with inhibition offering dual benefits—ameliorating both metabolic dysregulation and inflammation‐driven tissue damage [45].

PCSK9 exerts immunomodulatory effects beyond lipid metabolism by engaging innate and adaptive immune pathways. It activates the TLR4–SAPK/JNK signaling axis, which is frequently associated with chronic inflammation and autoimmune conditions. Moreover, PCSK9 downregulates monocyte CCR2, potentially attenuating leukocyte migration to inflamed tissues—a mechanism aligned with studies where CCR2 blockade ameliorates inflammation in atherosclerotic and autoimmune models.

Notably, PCSK9 upregulates MHC‐I expression in tumor cells, enhancing antigen presentation and supporting synergistic potential with immunotherapies in oncology. Its correlation with CRP in SLE underscores its relevance as an immune‐metabolic biomarker and therapeutic target.

Critical unresolved questions involve tissue‐specific mechanisms of PCSK9–immune interactions and its impact on regulatory T cells and tolerogenic dendritic cells. Future investigations should evaluate PCSK9‐i in IL‐17‐ or TNF‐α‐driven autoimmune pathologies, such as rheumatoid arthritis and psoriasis. Integrated multiomics approaches may elucidate novel lipid–immune crosstalk, advancing precision immunotherapies targeting PCSK9.

## 5 Clinical Translation and Trial Evidence

PCSK9‐i are now established as a cornerstone of intensive lipid‐lowering therapy in ASCVD. They consistently reduce LDL‐C levels by 59–77% and lower ischemic stroke risk by 21–27%, while demonstrating a favorable safety profile across diverse populations, including those with chronic kidney disease (CKD), diabetes, and East Asian ancestry (Table 2) [133, 134, 135, 136, 137]. Beyond these foundational benefits, emerging evidence supports their role in plaque regression and acute‐phase management, further expanding their clinical utility.

**TABLE 2:** Clinical characteristics and number of strokes reported in each.

Trial	NTC	Study Drug	Number of PCSK treated	Number of controls	Age	DM (%)	HT (%)	Smoking (%)	Number of strokes	
									Treated	Controls
OSLER I‐II	01439880	Evolocumab	2976	1489	58.1	13.0	52	15.5	1	3
ODYSSEY long term	01507831	Alirocumab	1553	788	60.4	34.5	−	20.5	9	2
Glagov	01813422	Evolocumab	484	484	59.8	20.5	83	23.3	2	3
FOURIER	01764633	Evolocumab	13, 784	13, 780	62.5	36.5	80.0	28.3	207	262
SPIRE I‐II	01968980	Bococizumab	13, 720	13, 718	63.3	47.5	80.8	24.5	45	75
ODYSSEY	01954394	Alirocumab	9462	9462	58.5	29.0	65.0	24.0	111	152
Total:	−	−	41, 979	39, 721	60.5	32.1	69.5	22.6	375	497

*Note*: Clinical trial.

### 5.1 Efficacy in Lipid‐Lowering and Cardiovascular Outcomes (MACE)

#### 5.1.1 Synergistic LDL‐C Suppression and Stroke Risk Mitigation

PCSK9‐i demonstrate robust efficacy in reducing LDL‐C levels and mitigating MACEs, particularly ischemic stroke. The FOURIER trial (evolocumab), a landmark study involving 27, 564 patients with established ASCVD, achieved a 59% reduction in LDL‐C and a 21% lower risk of ischemic stroke (HR 0.79, 95% CI 0.66–0.95), with sustained benefits persisting through extended follow‐up periods [49, 138]. This aligns with findings from the ODYSSEY Outcomes trial (alirocumab), which enrolled 18, 924 patients post‐ASC. Alirocumab reduced stroke incidence by 27% (HR 0.73, 95% CI 0.57–0.93), while exhibiting no increased risk of hemorrhagic stroke (HR 0.83, 95% CI 0.42–1.65). Notably, this cerebroprotective effect remained consistent across high‐risk subgroups, including patients with baseline LDL‐C ≥ 100 mg/dL or prior cerebrovascular events, over a median follow‐up of 2.8 years [134, 139].

The mechanistic basis for stroke protection may extend beyond LDL‐C reduction alone. PCSK9‐i modulate inflammation pathways (e.g., reduced IL‐6 and hsCRP levels) and stabilize atherosclerotic plaques, thereby attenuating thromboembolic events originating from carotid or intracranial arteries. This dual action—lipid‐lowering and plaque stabilization—positions PCSK9‐i as uniquely effective in cerebrovascular risk mitigation.

Long‐term efficacy in LDL‐C control was further substantiated by the ODYSSEY LONG TERM trial. Adjunctive alirocumab therapy added to maximally tolerated statins achieved sustained LDL‐C reductions of 62% versus placebo (*p* < 0.001) over 78 weeks [140]. Subsequent posthoc analyses revealed a 15% relative risk reduction in MACE (composite of CV death, MI, or stroke), underscoring the correlation between profound LDL‐C lowering and cardiovascular risk attenuation.

In patients with refractory hypercholesterolemia, the ODYSSEY COMBO I/II trials demonstrated that alirocumab added to high‐intensity statin plus ezetimibe therapy achieved incremental LDL‐C reductions of 45–50% from baseline. This highlights its role in addressing unmet needs in lipid management, particularly where conventional therapies fail to achieve guideline‐directed LDL‐C targets [141].

### 5.2 Exploratory Insights Into Pleiotropic Effects in Humans

#### 5.2.1 Determinants of Response: From Genetic Dyslipidemias to Metabolic Comorbidities

Homozygous FH (HoFH), a genetic disorder characterized by markedly elevated LDL‐C levels from birth, predisposes individuals to early‐onset and progressive ASCVD. Early therapeutic intervention is crucial for mitigating cardiovascular risk; however, many patients fail to achieve guideline‐recommended LDL‐C targets.

In the TAUSSIG trial (NCT01624142) evaluating evolocumab, a 23% reduction in LDL‐C was observed among HoFH patients, with a favorable safety profile [142, 143, 144]. By contrast, the ORION‐5 randomized clinical trial (NCT03851705) assessing inclisiran in HoFH demonstrated that despite significant reductions in PCSK9 levels, inclisiran therapy did not significantly lower LDL‐C concentrations in the adult HoFH cohort [145]. Nonetheless, inclisiran exhibited good tolerability, with safety findings consistent with prior studies. Conversely, Part 1 of the ORION‐13 study indicated that over 1 year, inclisiran effectively reduced LDL‐C in adolescent HoFH patients and was well tolerated [146]. These results suggest inclisiran may serve as a potential adjunctive therapy for adolescents with HoFH and minimal residual LDLR activity.

Beyond LDL‐C reduction, PCSK9 inhibition therapy may favorably influence cholesterol clearance kinetics in heterozygous FH (HeFH). In CKD populations, subgroup analyses from the FOURIER‐CKD study revealed preserved cardiovascular risk reduction benefit and acceptable safety with evolocumab in patients with estimated glomerular filtration rate (eGFR) <60 mL/min/1.73 m^2^. Findings from the ODYSSEY DM‐INSULIN and DM‐DYSLIPIDEMIA trials demonstrated significant LDL‐C lowering with alirocumab in diabetic patients, without significant alteration in glycemic control [147, 148].

#### 5.2.2 Time‐Sensitive Intervention: From Plaque Morphology to Guideline‐Directed Therapy

Acute‐phase intervention studies, such as the EVOPACS trial, validated the safety and feasibility of rapid lipid‐lowering postevent, supporting early intensive therapy. Imaging studies like Huygens and PACMAN‐AMI further demonstrated that acute‐phase PCSK9 inhibitor treatment stabilizes atherosclerotic plaques, reducing lipid‐rich necrotic core volume and enhancing fibrous cap thickness [149].

The 2022 American Heart Association (AHA) Scientific Statement advocates for early intensive lipid‐lowering therapy incorporating PCSK9‐i in extremely high‐risk ASCVD populations. Complementarily, the 2023 ESC/EAS Guidelines assign Class I recommendations for PCSK9 inhibitor initiation in high‐risk ASCVD patients failing to achieve LDL‐C targets despite maximally tolerated statin plus ezetimibe therapy [150].

The FITTER trial—a multicenter, randomized, double‐blind, placebo‐controlled study—assessed PCSK9 inhibitor efficacy in statin‐treated ACS patients with multivessel disease. This investigation demonstrated substantial reductions in LDL‐C concentrations alongside significant improvements in coronary microvascular function within this high‐risk cohort [151].

#### 5.2.3 Exploratory and Analytical Research Breakthroughs

OSLER‐1/2 (evolocumab) showed that no new safety signals were identified during more than 7 years of follow‐up. The FOURIER‐OLE results showed that after 5.5 years of continuous treatment, LDL‐C remained below 30 mg/dL, and cardiovascular benefits increased with prolonged treatment duration [64]. Additionally, the HEYMANS study (a prospective registry enrolling adults from 12 European countries who initiated evolocumab treatment in routine clinical practice) demonstrated that evolocumab treatment was associated with sustained reductions in LDL‐C levels for up to 30 months, and that the persistence of evolocumab was high at both 12 and 30 months. Expanding the use of monoclonal antibodies such as evolocumab can improve LDL‐C control at the population level and confirm safety outcomes consistent with randomized controlled trials (RCTs) [152, 153, 154].

Lipid reduction and fibrous cap thickening directly mitigate plaque rupture risk, constituting the pathophysiological mechanism underlying reduced cardiovascular events. Regarding cerebrovascular benefits, the PACMAN‐AMI trial (NCT03067844) utilizing evolocumab demonstrated significant coronary plaque regression via serial intravascular ultrasound (IVUS) assessment after 52 weeks. This landmark study constitutes the first multimodal imaging verification that PCSK9 inhibitor–statin combination therapy induces substantial plaque regression in nonculprit coronary arteries [97, 155, 156, 157, 158]. At the lesion‐specific level, intensive lipid‐lowering yielded >twofold greater regression in high‐risk vulnerable plaques compared with whole‐vessel analyses, providing compelling evidence for targeted therapy in high‐risk cohorts [159]. For secondary stroke prevention, posthoc analyses of the discontinued SPIRE program (investigating bococizumab, which exhibited neutralizing antibody‐mediated immunogenicity) suggested potential reduction in recurrent stroke risk [160]. Concurrently, the ongoing ORION‐4 trial (NCT03705234) is evaluating inclisiran's siRNA‐mediated PCSK9 suppression to determine its impact on recurrent cerebrovascular events‐findings that may support biannual administration of this RNA therapeutic [161].

#### 5.2.4 Neuroprotective Disconnect across Species: BBB Impermeability and Molecular Size Constraints

Despite demonstrating pronounced neuroprotection in preclinical models—including significant reductions in cerebral infarct volume and improved neurological deficit scores—these findings exhibit limited translatability to human stroke populations [162, 163]. Most experimental paradigms utilize young, healthy transgenic rodents subjected to induced cerebral ischemia, failing to recapitulate clinical comorbidities (e.g., diabetes mellitus, chronic hypertension) or heterogeneous stroke etiologies (e.g., cardioembolic, lacunar) prevalent in human cohorts [164, 165, 166]. Additionally, therapeutic intervention windows in animal studies (typically pre‐ or peri‐ischemic administration) diverge substantially from clinical realities of delayed treatment initiation [167].

BBB impermeability constitutes a fundamental translational barrier, potentially restricting central nervous system bioavailability. The substantial molecular mass (∼150 kDa) of monoclonal antibody‐based PCSK9‐i (e.g., evolocumab) precludes passive diffusion across the BBB, likely limiting direct neuroprotective effects [168, 169]. While novel RNA interference therapeutics (e.g., inclisiran) offer extended pharmacodynamic profiles, their anionic macromolecular structure similarly impedes BBB transit [170, 171].

Bridging this translational gap necessitates: integrated analyses of postmortem human neurovascular tissue; dedicated clinical trials in cerebrovascular disease populations; cross‐species comparative pharmacodynamic studies. These approaches will elucidate the therapeutic relevance of PCSK9 inhibition in stroke pathophysiology.

## 6 Clinical Applications and Protocol Development

Studies demonstrate that in‐hospital initiation of PCSK9‐i within 72 h of ACS rapidly reduces LDL‐C by 50–60%, improves lipid target attainment, and lowers 30‐day MACE risk by 28%, with no significant safety concerns [172]. The 2023 AHA/ASA Guidelines formalize this approach, recommending PCSK9‐i combined with maximally tolerated statins for very high‐risk ischemic stroke patients with LDL‐C ≥70 mg/dL. This recommendation synthesizes evidence from pivotal trials, including: FOURIER trial: 59% LDL‐C reduction and 21% ischemic stroke risk reduction with evolocumab, SPARCL study: validation of intensive lipid‐lowering for secondary stroke prevention, 2021 AHA/ASA update: emphasis on LDL‐C thresholds for advanced therapies.

## 7 Challenges, Unmet Needs, and Future Perspectives

### 7.1 Translational Gaps and Limitations in Current Models

#### 7.1.1 Differential Anti‐Inflammatory Effects at Systemic versus Tissue Levels in Humans

Multiple clinical trials demonstrate that PCSK9‐i exert minimal or negligible effects on reducing circulating inflammatory markers, such as hs‐CRP [138, 173, 174, 175]. Despite achieving substantial reductions in LDL‐C of 55–60%, these agents were associated with only a modest, nonsignificant decrease in hs‐CRP of 3.2% (95% CI: −8.1 to +1.7%; *p* = 0.21) [176, 177, 178, 179, 180, 181]. This dissociation between LDL‐C lowering and anti‐inflammatory effects was further investigated in the large‐scale SPIRE trial. Utilizing proteomic analysis, a dedicated sub‐study comprehensively assessed 92 plasma inflammatory proteins, including key cytokines like IL‐6 and TNF‐α [36]. The findings revealed that these inflammatory markers failed to exhibit significant alterations following PCSK9 inhibitor treatment, despite pronounced LDL‐C reduction. These data collectively suggest that the primary mechanism underlying the antiatherosclerotic efficacy of PCSK9‐i is robust lipid lowering, rather than direct modulation of inflammatory pathways. This view is reinforced by results from the PROMISE study, which highlighted that patients at high inflammatory risk (defined by hs‐CRP ≥ 3 mg/L) experienced a significantly elevated risk of cardiovascular events, even when target LDL‐C levels were successfully achieved. Regarding the observed biological dissociation between hs‐CRP and LDL‐C, a mechanistic explanation proposes that hs‐CRP, an established inflammatory biomarker, predominantly reflects the activation status of the IL‐6 signaling pathway [182]. In contrast, LDL‐C reduction via PCSK9 inhibition is primarily mediated by the upregulation of LDLRs on hepatocytes. These represent distinct, nonoverlapping pathophysiological processes [183, 184].

In vivo analyses demonstrated significant localized anti‐inflammatory effects within atherosclerotic plaques. A prespecified PACMAN‐AMI trial sub‐study revealed that alirocumab, a PCSK9 inhibitor, elicited significant reductions in plaque macrophage infiltration and inflammatory cytokine expression (notably IL‐6 and TNF‐α). Concurrently, it enhanced plaque stability through attenuated lipid core volume and increased fibrous cap thickness [61, 97, 185, 186].Comparative histopathological evaluation of carotid endarterectomy specimens further established that PCSK9 inhibitor‐treated patients exhibited significantly lower intraplaque inflammatory mediator levels versus statin‐treated counterparts, despite comparable achievement of guideline‐recommended LDL‐C targets (<70 mg/dL) [187, 188, 189]. Carotid MRI vasculature imaging corroborated these findings, demonstrating PCSK9 inhibitor therapy significantly suppressed plaque neovascularization and reduced concentrations of instability‐associated biomarkers, including osteopontin and MMPs [190, 191, 192, 193, 194].

Analysis revealed a significant localized anti‐inflammatory effect in the resolution of myocardial inflammation. The EVACS I/II trial demonstrated that early administration of the PCSK9 inhibitor evolocumab accelerates myocardial inflammatory resolution following ACS and may attenuate adverse cardiac remodeling, despite unaltered systemic inflammatory biomarkers [195, 196, 197]. Serum PCSK9 concentrations exhibit a positive correlation with myocardial inflammation severity. Mechanistically, PCSK9‐i suppress local NF‐κB activation through blockade of the CAP1–PKCδ signaling axis, thereby mitigating myocardial ischemia–reperfusion injury [198].

PCSK9‐i elicit significant local anti‐inflammatory effects on both vascular endothelial cells and smooth smooth muscle cells. Specifically, they effectively downregulate LOX‐1 receptor expression, attenuate oxLDL‐induced endothelial cell apoptosis and monocyte adhesion, thereby ameliorating vascular endothelial function [63]. Furthermore, studies employing animal models have demonstrated that PCSK9‐knockout or antibody‐mediated inhibition reduces macrophage infiltration and MMP‐9 expression within AAAs, inhibiting the degradation of elastic fibers in the vascular wall [36]. These findings indicate potent localized protective actions of PCSK9 inhibition on vascular biology.

The tissue‐restricted effects may originate from several distinct molecular mechanisms. First, PCSK9‐i significantly upregulate LDLR expression on plaque‐resident macrophages, thereby facilitating oxLDL efflux and clearance. This process attenuates NLRP3 inflammasome priming—an effect contingent upon achieving supraphysiologic local drug concentrations within atherosclerotic lesions, a pharmacokinetic profile not attainable during systemic circulation [41]. Second, these inhibitors potentially mitigate mitochondrial oxidative stress in plaque endothelial cells via SIRT3 upregulation, subsequently suppressing downstream NF‐κB signal transduction [42, 67]. This differential effect may account for the superior reduction in stroke risk observed with PCSK9 inhibition in the FOURIER trial, despite achieving LDL‐C levels comparable to those attained with other LDL‐lowering therapies. Future studies should use advanced imaging techniques to link PCSK9 inhibitor therapy with inflammatory changes in atherosclerotic plaques [199]. Additionally, investigating the durability of these tissue‐specific effects within high‐risk subgroups (e.g., individuals with diabetes mellitus) is warranted. The observed dissociation between systemic and local anti‐inflammatory responses underscores the imperative to develop atherosclerosis‐specific biomarkers that extend beyond conventional blood‐based assays.

#### 7.1.2 Unmet Need in Stroke Prevention: Subtype‐Specific Efficacy Challenges

Although PCSK9‐i have demonstrated definitive efficacy in reducing MACEs, their impact on stroke‐related outcomes remains incompletely elucidated. Large‐scale RCTs, namely FOURIER (evolocumab) and ODYSSEY OUTCOMES (alirocumab), were primarily powered to assess composite cardiovascular endpoints, incorporating stroke as a secondary or exploratory component [25, 27, 86]. Subsequent subgroup analyses within these trials have suggested a trend toward stroke risk reduction; however, these findings frequently lack statistical significance or have demonstrated marginal clinical impact relative to the primary outcome. This attenuation of effect on stroke prevention may be attributable, in part, to specific design limitations inherent in the existing trials.

Current clinical evidence on PCSK9‐i predominantly focuses on coronary artery disease populations, with limited representation of high‐risk stroke cohorts. Notably, only 15–20% of participants in major trials had a prior history of cerebrovascular events, while critical subgroups such as intracranial atherosclerotic stenosis (ICAS) patients—a population with inherently elevated stroke vulnerability—remain markedly underrepresented. This gap underscores a significant limitation in extrapolating cardiovascular outcomes to neurovascular prevention paradigms. The landmark 2025 ICAS prospective cohort study addressed this research void, demonstrating that adjunctive PCSK9 inhibitor therapy (combined with statins) achieved a 66.5% reduction in early recurrent stroke risk. Critically, the LDL‐C reduction was significantly more pronounced in the combination group, establishing a robust dose–response relationship between lipid‐lowering intensity and secondary stroke prevention efficacy in ICAS [200]. Second, the follow‐up durations in these trials, typically 2–3 years, may be insufficient to reliably detect a significant preventive effect on ischemic stroke. This timeframe could potentially be inadequate for manifesting the full impact of LDL‐C lowering via PCSK9‐i on atherosclerotic plaque stabilization and regression within the cerebrovascular circulation, processes hypothesized to evolve over a more protracted timeline than the stabilization observed in ACSs [201].

To date, no large‐scale RCTs have performed stratified outcome analyses based on stroke etiologic subtypes (TOAST classification) [173, 202]. Existing studies have failed to demonstrate a significant preventive efficacy of PCSK9 inhibitors for either cardioembolic or small‐vessel occlusion (lacunar) stroke. Pooled analyses indicate that PCSK9 inhibition may lower the risk of large‐artery atherosclerotic stroke—an effect attributable to its LDL‐C‐lowering mechanism—but exhibits no discernible effect on cardioembolic or small‐vessel strokes. Furthermore, persistent concerns remain that achieving very low LDL‐C concentrations (<25 mg/dL [<0.65 mmol/L]) may elevate hemorrhagic stroke risk, a signal identified in the FOURIER trial [114]. However, the limited number of events precludes definitive conclusions regarding this potential association.

In conclusion, while there is biological plausibility supporting a role for PCSK9‐i in stroke prevention, definitive evidence of clinically significant benefit requires dedicated outcome trials with prolonged follow‐up enrolling high‐risk cohorts. Presently, the primary indication for these agents remains cardiovascular risk reduction, with insufficient evidence to support a stroke‐specific protective effect.

The role of PCSK9‐i in stroke prevention necessitates a paradigm shift from a “lipid‐lowering‐centric” approach to a “multimodal synergistic” strategy. Current evidence supports significant benefits in specific high‐risk populations (e.g., symptomatic ICAS or elevated lipoprotein(a) [Lp(a)] levels), yet their ineffectiveness in nonatherosclerotic strokes (such as cardioembolic events) underscores the need for individualized therapy. Future research should focus on validating long‐term safety and clinical translational value of their pleiotropic effects through dedicated RCTs, while concurrently developing predictive models to optimize treatment cost effectiveness.

#### 7.1.3 Limitations of PCSK9‐i in Translating to Human Stroke Pathophysiology

Current evidence does not establish a cerebrovascular‐specific mechanism for PCSK9‐i in human stroke. Research on PCSK9 in atherosclerosis primarily stems from coronary or peripheral vascular studies. However, the cerebrovascular microenvironment—including the BBB and local inflammation—may differ significantly [203]. Regarding carotid plaque mechanisms, studies ex vivo using human carotid plaques demonstrate that PCSK9 induces mitochondrial fission via the p38–DRP1 pathway, promoting VSMC apoptosis and plaque vulnerability. This finding lacks direct validation in cerebral arteries [55, 204]. PCSK9 also exacerbates cerebrovascular endothelial inflammation and oxidative stress via the LOX‐1 receptor, but whether this regulatory mechanism aligns with coronary arteries remains unclear [205]. Future research should address these gaps through cross‐organ cellular studies, molecular analyses of intracranial plaques, and targeted animal models to determine the pathway's universality and therapeutic potential in cerebral arterial disease.

Regarding translational gaps in neuroprotection, animal studies suggest PCSK9‐i may attenuate postischemic neuronal apoptosis by downregulating ApoER2. However, human stroke involves more complex neuroinflammation and glial responses, while existing studies have not distinguished between stroke subtypes (e.g., embolic versus thrombotic) [124, 206, 207]. Future research requires molecular subtyping studies across stroke subtypes, integrated with multiomics approaches (e.g., single‐cell sequencing) to elucidate the regulatory networks of neuroinflammation and glial responses, thereby advancing precision neuroprotective strategies.

A key limitation is the lack of robust human data demonstrating a direct effect of PCSK9‐i on stroke‐specific outcomes. Although large cardiovascular outcome trials (e.g., FOURIER, ODYSSEY) reported reductions in composite endpoints including stroke, they were not designed to isolate stroke mechanisms or assess neuroprotective effects. Furthermore, no definitive evidence currently demonstrates PCSK9 inhibitor efficacy in hemorrhagic stroke, and theoretical concerns persist that very low LDL‐C levels (<30 mg/dL) may increase intracranial hemorrhage risk—a signal noted in some trials requiring further investigation. Future research necessitates randomized trials with neuroprotection as a primary endpoint, integrated with multiomics approaches for mechanistic insights.

The mechanisms by which PCSK9‐i affect stroke pathophysiology require further clinical validation. While the multiple effects of PCSK9‐i (e.g., anti‐inflammatory and antioxidant properties) show promise in vitro and in animal models, conclusive human evidence is still lacking. Some patients exhibit reduced plaque lipid core post‐PCSK9 inhibitor administration without improved inflammatory markers, suggesting insignificant anti‐inflammatory effects [138, 208]. LOF mutations (e.g., R46L) lower LDL‐C and ischemic stroke risk, but distinguishing direct cerebrovascular protection from systemic lipid‐lowering effects remains challenging [209, 210, 211].

Research reliance on surrogate endpoints (e.g., cIMT, cfPWV) rather than direct stroke outcomes highlights biomarker limitations [212, 213]. The lack of dedicated stroke trials evaluating imaging biomarkers (e.g., carotid MRI‐assessed plaque stability) or mechanistic endpoints (e.g., microglial activation) impedes clinical translation [214, 215, 216]. Bridging this gap requires multiomics data integration (e.g., metabolomics + proteomics + imaging) via machine learning and adaptive trial designs (e.g., umbrella trials) to validate composite endpoints.

### 7.2 Novel Therapeutic Technologies and Drug Development

#### 7.2.1 Small‐Molecule PCSK9 Antagonism: Clinical‐Stage Oral Delivery Advancement

AZD0780 (AstraZeneca), a small molecule targeting PCSK9–LDLR interactions, demonstrated dose‐dependent efficacy in the phase II PURSUIT trial. At the 30 mg dose combined with statins, it achieved a 50.7% reduction in LDL‐C over 12 weeks, highlighting its potential for integration into conventional lipid‐lowering regimens [217]. MK‐0616, another oral small molecule inhibitor, has shown promising results across early trials: phase I data confirmed its safety and tolerability, while phase IIb trials revealed a 60.9% LDL‐C reduction at the 30 mg dose. This robust efficacy has propelled MK‐0616 into global phase III clinical testing, positioning it as a frontrunner in oral PCSK9 inhibition [218, 219].

Simultaneously, CVI‐LM001, the first‐in‐class small molecule inhibitor targeting hepatocyte nuclear factor‐1α to indirectly modulate PCSK9 expression, has entered phase II trials. Its unique mechanism bypasses direct PCSK9 binding, potentially reducing off‐target effects while maintaining significant LDL‐lowering activity [218].

#### 7.2.2 Epigenome Editing for Sustained Silencing: Lipid Nanoparticle‐Delivered Methylation Therapy

PCSK9‐EE (developed by Chroma Medicine et al.) utilizes lipid nanoparticles to deliver an epigenetic editor—a DNMT3A/dCas9 complex—that specifically targets the PCSK9 promoter region. This innovative approach induces DNA methylation at the promoter site, achieving durable gene silencing and sustained LDL‐C reduction. In preclinical studies, a refined candidate, PCSK9‐EE‐V2, demonstrated optimal activity and persistence in nonhuman primates, maintaining >70% PCSK9 suppression for over 12 months posttreatment. Further optimization of delivery specificity and safety profiles is ongoing to advance clinical translation [167].

#### 7.2.3 Adherence‐Optimized Synergy: Infrequent Dosing With Complementary Mechanisms

**FIGURE 4:**
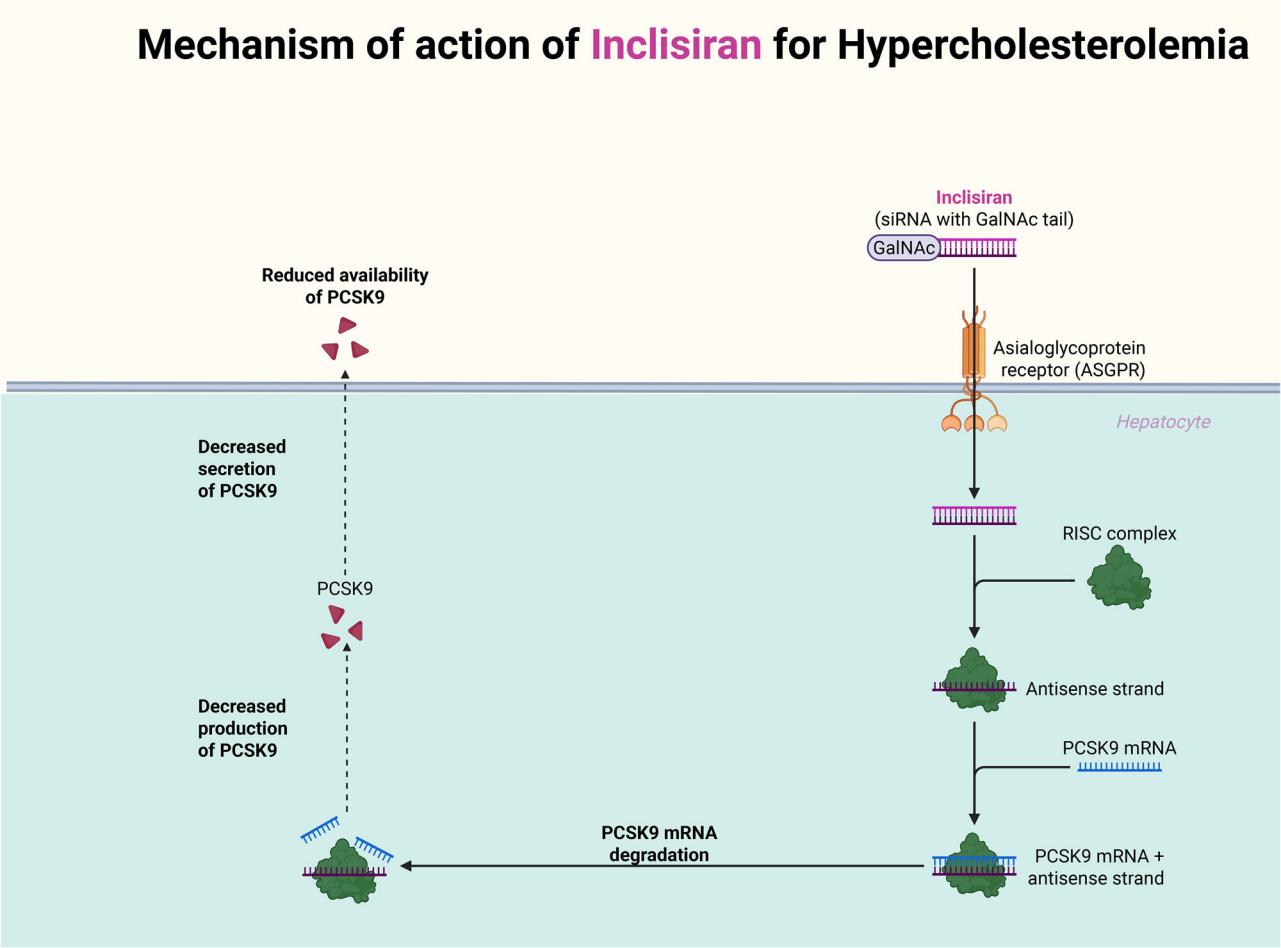
Mechanism of action of inclisiran. Inclisiran is a first‐in‐class synthetic small‐interfering RNA (siRNA) therapeutic designed to specifically target and silence the PCSK9 gene in hepatocytes. Its mechanism involves a sophisticated RNA interference (RNAi) pathway: after subcutaneous administration, inclisiran is transported to the liver where it binds to the RNA‐induced silencing complex (RISC). The activated RISC then selectively cleaves PCSK9 messenger RNA (mRNA), preventing its translation into functional PCSK9 protein. By inhibiting PCSK9 synthesis at the genetic level, inclisiran upregulates hepatic LDL receptors (LDLR) that would otherwise be degraded by PCSK9. This leads to enhanced LDLR recycling on hepatocyte surfaces, significantly improving LDL‐cholesterol (LDL‐C) clearance from the bloodstream. The RNAi‐mediated effect provides prolonged pharmacological activity, with a single dose achieving >50% reduction in circulating PCSK9 and LDL‐C levels for 3–6 months.

The development of long‐acting siRNA therapies, such as inclisiran, has opened new avenues for synergistic lipid management (Figure 4). When combined with statins or oral PCSK9‐i, inclisiran demonstrates the potential to achieve LDL‐C reductions exceeding 80%, leveraging its biannual dosing regimen to sustain profound lipid‐lowering effects. For instance, the AZD0780 (oral PCSK9 inhibitor) and rosuvastatin combination, evaluated in a phase I trial, reduced LDL‐C by an additional 51% compared with statin monotherapy, highlighting the potent additive effects of dual‐mechanism approaches. Emerging data suggest such combinations not only enhance efficacy but may also improve medication adherence by reducing dosing frequency. However, long‐term safety and cost effectiveness of these regimens require further validation in larger, diverse patient populations [217].

#### 7.2.4 Disruptive Therapeutic Platforms: Base Editors and Antisense Oligonucleotides

**FIGURE 5:**
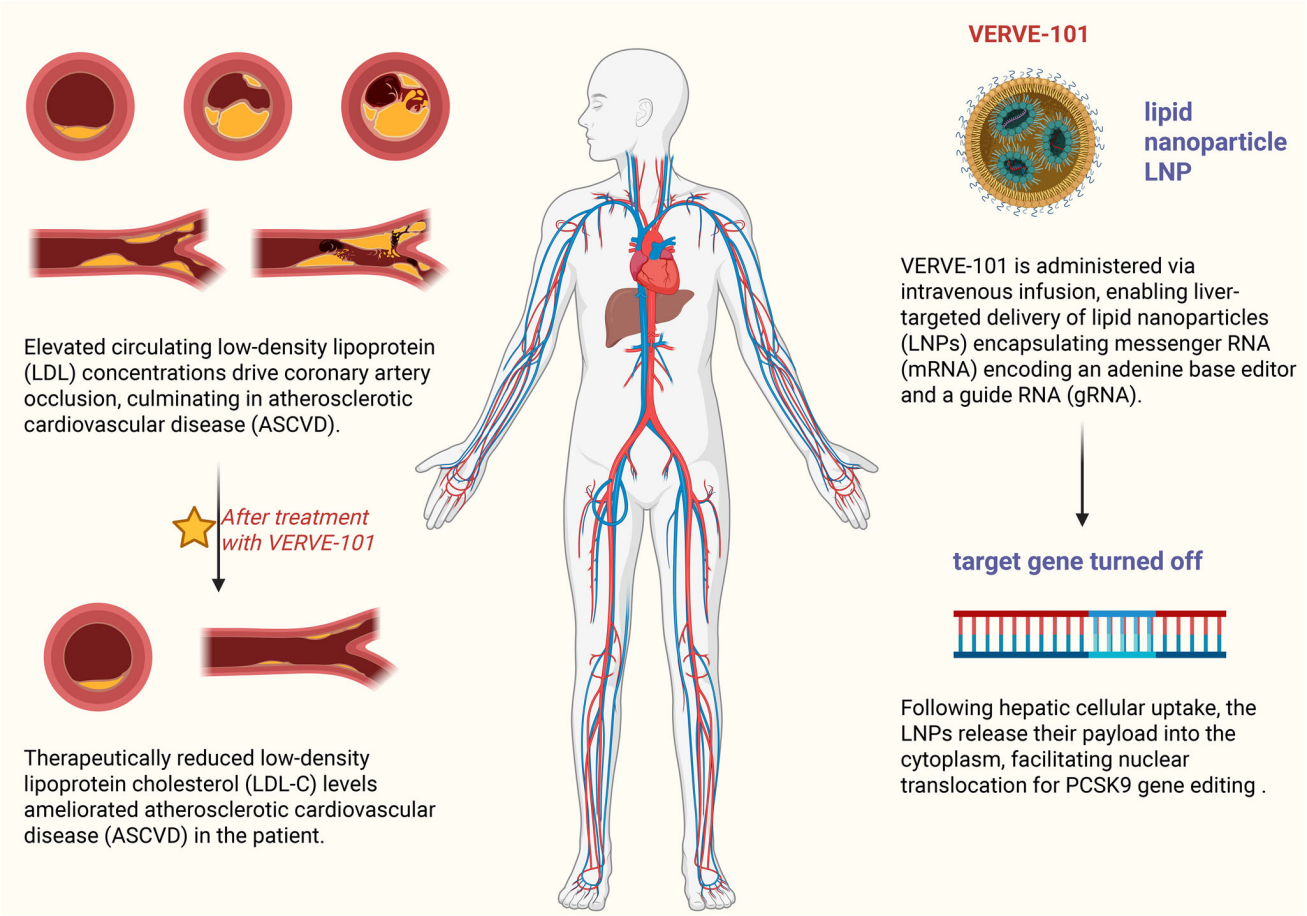
Mechanism of action of VERVE‐101. VERVE‐101 is an innovative base‐editing therapy designed to permanently inactivate the *PCSK9* gene in hepatocytes, offering a potential one‐time treatment for hypercholesterolemia. This CRISPR‐based therapy utilizes an adenine base editor (ABE) delivered via lipid nanoparticles (LNPs) to precisely convert a single adenine (A) to guanine (G) in the *PCSK9* gene, creating a premature stop codon that halts PCSK9 protein production. By permanently silencing *PCSK9* at the DNA level, VERVE‐101 upregulates hepatic LDL receptors (LDLR), mimicking natural loss‐of‐function mutations associated with lifelong low LDL‐C. Unlike RNAi therapies (e.g., inclisiran) requiring redosing, VERVE‐101's single‐administration approach enables durable *PCSK9* knockout, potentially providing lifelong LDL‐C reduction (>50%) and cardiovascular protection. The LNP delivery system ensures hepatocyte‐specific targeting, minimizing off‐target effects. Preclinical data show efficient *PCSK9* editing (>80%) with sustained LDL‐C lowering.

VERVE‐101, a pioneering base‐editing therapy, achieves permanent PCSK9 gene knockout in hepatocytes, demonstrating robust LDL‐C reduction (60%) in early animal trials (Figure 5). While promising, challenges persist in optimizing liver‐specific delivery and ensuring long‐term safety, particularly regarding off‐target genomic effects and immune responses to editing components [167]. Concurrently, pelacarsen, an antisense oligonucleotide targeting Lp(a), has shown remarkable efficacy in Phase II trials, reducing Lp(a) levels by 72–80% through selective degradation of apolipoprotein(a) [apo(a)] mRNA. This reduction correlates with attenuated inflammation and thrombotic risk, positioning pelacarsen as a potential game‐changer for patients with elevated Lp(a)‐driven cardiovascular risk [220].

### 7.3 Defining Future Research Agendas

#### 7.3.1 Multidimensional Knowledge Gaps: Therapeutic Innovation Barriers

Key knowledge gaps include defining the optimal dosing windows and dose optimization during acute‐phase treatment, as well as clarifying the long‐term safety profile, particularly regarding cognitive function impacts with sustained ultra‐low LDL‐C levels (<20 mg/dL). Advancing novel formulations—such as oral PCSK9‐i or ultra‐long‐acting injectables—remains critical to improve adherence, while emerging approaches like PCSK9 vaccines and gene‐editing therapies (e.g., VERVE‐101) aim to achieve durable benefits through single‐course interventions.

Precision medicine strategies are being developed to identify high‐risk patients through predictive models incorporating ALT levels, baseline LDL‐C, and statin responsiveness. Concurrently, combination therapies targeting residual risks—such as pairing PCSK9‐i with Lp(a)‐lowering RNA therapies (e.g., pelacarsen)—are under investigation to address multifaceted lipid and inflammatory pathways.

Basic research priorities include elucidating the role of PCSK9 in acute ischemic stroke and intracranial artery stenosis, as well as exploring synergistic regimens combining PCSK9‐i with statins and antiplatelet agents (e.g., aspirin) to achieve multiple effects through lipid‐lowering, anti‐inflammatory, and antithrombotic effects. Indication expansion efforts focus on clarifying PCSK9's platelet CD36 receptor interactions and nonlipid pathways to develop next‐generation therapies targeting novel mechanisms (e.g., CAP1 inhibition). Large‐scale RCTs are urgently needed to validate PCSK9‐i’ efficacy in primary prevention of VTE and other noncardiovascular indications. Addressing these challenges will require interdisciplinary collaboration to translate mechanistic insights into transformative clinical applications.

#### 7.3.2 Resource Allocation Efficiency: Cost‐Accessibility Disparities in Stroke Prophylaxis

Despite the demonstrated efficacy of PCSK9‐i in reducing LDL‐C and cardiovascular events, their widespread adoption for stroke prevention faces significant financial and operational barriers. High treatment cost remains the primary obstacle, with annual per‐patient expenses substantially exceeding those of generic statins [221, 222, 223]. Cost‐effectiveness debates persist, and the high cost drives stringent prior authorization requirements (US rejection rates: 40–60%), increasing administrative burdens [224, 225].

Accessibility challenges further impede implementation. Utilization is highly inequitable, with over 80% concentrated in high‐income countries. Low‐ and middle‐income countries face prohibitive costs and lack infrastructure for monoclonal antibody injection, while remote areas encounter additional barriers [226, 227, 228]. Patient adherence is suboptimal due to the requirement for biweekly subcutaneous injections compared with oral therapies [229].

Innovative solutions are being explored. Stroke guidelines recommend PCSK9‐i use concomitant with healthcare payment reforms to ensure equitable access. Current economic thresholds restrict PCSK9‐i use primarily to high‐risk patients within well‐resourced systems, despite their therapeutic potential. Future research should focus on: real‐world data‐based pharmacoeconomic evaluations in stroke‐specific populations and developing risk‐stratification tools to optimize targeted therapy.

## 8 Conclusion

PCSK9‐i have demonstrated significant therapeutic potential across the spectrum of ischemic stroke management, encompassing primary prevention, secondary prevention, acute‐phase treatment, and long‐term prognosis improvement. Their multifaceted mechanisms of action—including LDL‐C reduction, stabilization of atherosclerotic plaques via anti‐inflammatory effects, attenuation of thrombotic risk, and endothelial function restoration—position these agents as versatile tools for addressing both the metabolic and inflammatory drivers of cerebrovascular disease. Clinical trials such as FOURIER and ODYSSEY Outcomes have validated their capacity to reduce ischemic stroke risk by 21–27% while maintaining a favorable safety profile, even in high‐risk populations with comorbid conditions.

Despite these advances, limitations persist in current research and clinical application. The long‐term cognitive effects of sustained LDL‐C lowering to very low levels (<20 mg/dL) remain under investigation, and optimal timing for acute‐phase intervention requires further refinement. Additionally, cost effectiveness and accessibility challenges hinder widespread adoption in resource‐limited settings. Future high‐quality studies are needed to elucidate the full scope of PCSK9‐i’ multiple benefits, establish standardized treatment protocols for vulnerable plaque phenotypes, and explore synergistic effects with emerging therapies like antiplatelet agents or Lp(a)‐targeted drugs.

With ongoing research into novel formulations (e.g., oral PCSK9‐i, siRNA therapies) and biomarker‐guided approaches, PCSK9‐i are poised to become cornerstone therapies in ischemic stroke management. Their unique ability to concurrently target lipid metabolism, vascular inflammation, and thrombotic pathways offers unprecedented opportunities to improve clinical outcomes and quality of life for stroke survivors, ultimately reducing the global burden of cerebrovascular disease.

Simultaneously, the documented adverse effect profile of PCSK9‐i warrants consideration. While these agents lack association with statin‐class adverse effects such as hepatic dysfunction, new‐onset diabetes mellitus, rhabdomyolysis, or significant ophthalmic complications, they may elicit localized or systemic reactions. These include injection site discomfort (e.g., myalgia), cephalgia, and influenza‐like symptoms. Furthermore, although certain investigations suggest potential associations with increased hemorrhagic risk and altered neurocognitive function, the current evidence base remains insufficient. Definitive confirmation of these relationships necessitates further prospective, large‐scale studies.

As the therapeutic evidence evolves, PCSK9‐i demonstrate significant potential as an adjunctive or alternative therapeutic strategy in the secondary prevention of ischemic stroke. Their integration into management algorithms holds promise for substantially improving clinical outcomes in this patient population.

## Author Contributions

Jia Kuang and Lei Hao participated in writing. Zhao Yang participated in reviewing and editing. All authors have read and approved the final manuscript.

## Conflicts of Interest

The authors declare no conflicts of interest.

## Ethics Statement

No ethical approval was required for this study.

## Data Availability

The authors have nothing to report.

## References

[1] D. Sims , “Ask the Consultant: Stroke, ” Bmj 389 (2025): r436. 40246315 10.1136/bmj.r436

[2] V. L. Feigin , M. Brainin , B. Norrving , et al., “World stroke organization (WSO): Global stroke Fact Sheet 2022, ” International Journal of Stroke 17 (2022): 18–29. 34986727 10.1177/17474930211065917

[3] F. Mach , C. Baigent , A. L. Catapano , et al., “2019 ESC/EAS Guidelines for the Management of Dyslipidaemias: Lipid Modification to Reduce Cardiovascular Risk, ” European Heart Journal 41 (2020): 111–188. 31504418 10.1093/eurheartj/ehz455

[4] P. Gargiulo , C. Basile , A. Cesaro , et al., “Efficacy, Safety, Adherence and Persistence of PCSK9 Inhibitors in Clinical Practice: A Single Country, Multicenter, Observational Study (AT‐TARGET‐IT), ” Atherosclerosis 366 (2023): 32–39. 36696749 10.1016/j.atherosclerosis.2023.01.001

[5] V. L. Feigin , B. A. Stark , C. O. Johnson , et al., “Global, Regional, and National Burden of Stroke and Its Risk Factors, 1990–2019: A Systematic Analysis for the Global Burden of Disease Study 2019, ” Lancet Neurology 20 (2021): 795–820. 34487721 10.1016/S1474-4422(21)00252-0 PMC8443449

[6] J. Vicente‐Valor , X. García‐González , S. Ibáñez‐García , et al., “PCSK9 inhibitors Revisited: Effectiveness and Safety of PCSK9 inhibitors in a Real‐life spanish Cohort, ” Biomedicine and Pharmacotherapy 146 (2022): 112519. 34968928 10.1016/j.biopha.2021.112519

[7] X. Bao , Y. Liang , H. Chang , et al., “Targeting Proprotein Convertase Subtilisin/Kexin Type 9 (PCSK9): From Bench to Bedside, ” Signal Transduction and Targeted Therapy 9 (2024): 13. 38185721 10.1038/s41392-023-01690-3 PMC10772138

[8] H. H. Hobbs , J. C. Cohen , and J. D. Horton , “PCSK9: From Nature's Loss to Patient's Gain, ” Circulation 149 (2024): 171–173. 38227713 10.1161/CIRCULATIONAHA.123.064498 PMC10874118

[9] R. D. Santos , K. Nasir , and M. D. Shapiro , “A Step Forward for Long‐acting PCSK9 Inhibition: Improvements Without a Breakthrough, ” Journal of the American College of Cardiology 84 (2024): 2048–2050. 39387762 10.1016/j.jacc.2024.09.019

[10] T. E. Strandberg , P. T. Kovanen , D. M. Lloyd‐Jones , F. J. Raal , R. D. Santos , and G. F. Watts , “Drugs for Dyslipidaemia: The Legacy Effect of the scandinavian Simvastatin Survival Study, ” Lancet 404, no. 4S (2024): 2462–2475. 39577453 10.1016/S0140-6736(24)02089-0

[11] S. Hummelgaard , J. P. Vilstrup , C. Gustafsen , S. Glerup , and K. Weyer , “Targeting PCSK9 to Tackle Cardiovascular Disease, ” Pharmacology and Therapeutics 249 (2023): 108480. 37331523 10.1016/j.pharmthera.2023.108480

[12] N. G. Seidah and A. Prat , “The Biology and Therapeutic Targeting of the Proprotein Convertases, ” Nature Reviews Drug Discovery 11 (2012): 367–383. 22679642 10.1038/nrd3699

[13] S. Poirier , G. Mayer , S. Benjannet , et al., “The Proprotein Convertase PCSK9 Induces the Degradation of Low Density Lipoprotein Receptor (LDLR) and Its Closest family Members VLDLR and ApoER2, ” Journal of Biological Chemistry 283 (2008): 2363–2372. 18039658 10.1074/jbc.M708098200

[14] A. Grefhorst , M. C. McNutt , T. A. Lagace , and J. D. Horton , “Plasma PCSK9 Preferentially Reduces Liver LDL Receptors in Mice, ” Journal of Lipid Research 49 (2008): 1303–1311. 18354138 10.1194/jlr.M800027-JLR200 PMC2386900

[15] Y. Wang , Y. Huang , H. H. Hobbs , and J. C. Cohen , “Molecular Characterization of Proprotein Convertase Subtilisin/Kexin Type 9‐mediated Degradation of the LDLR, ” Journal of Lipid Research 53 (2012): 1932–1943. 22764087 10.1194/jlr.M028563 PMC3413232

[16] E. Gallego‐Colon , A. Daum , and C. Yosefy , “Statins and PCSK9 Inhibitors: A New Lipid‐lowering Therapy, ” European Journal of Pharmacology 878 (2020): 173114. 32302598 10.1016/j.ejphar.2020.173114

[17] J. Cohen , A. Pertsemlidis , I. K. Kotowski , R. Graham , C. K. Garcia , and H. H. Hobbs , “Low LDL Cholesterol in Individuals of african Descent Resulting From Frequent Nonsense Mutations in PCSK9, ” Nature Genetics 37 (2005): 161–165. 15654334 10.1038/ng1509

[18] I. K. Kotowski , A. Pertsemlidis , A. Luke , et al., “A Spectrum of PCSK9 Alleles Contributes to Plasma Levels of Low‐density Lipoprotein Cholesterol, ” American Journal of Human Genetics 78 (2006): 410–422. 16465619 10.1086/500615 PMC1380285

[19] M. Abifadel , M. Varret , J. Rabès , et al., “Mutations in PCSK9 Cause Autosomal Dominant Hypercholesterolemia, ” Nature Genetics 34 (2003): 154–156. 12730697 10.1038/ng1161

[20] M. S. Sabatine , J. A. Underberg , M. Koren , and S. J. Baum , “Focus on PCSK9 Inhibitors: From Genetics to Clinical Practice, ” Postgraduate Medicine 128, no. Suppl 1 (2016): 31–39. 27422124 10.1080/00325481.2016.1208895

[21] F. J. Raal , V. Mehta , M. Kayikcioglu , et al., “Lerodalcibep and evolocumab for the Treatment of Homozygous Familial Hypercholesterolaemia With PCSK9 Inhibition (LIBerate‐HoFH): A Phase 3, Randomised, Open‐label, Crossover, Non‐inferiority Trial, ” The Lancet Diabetes and Endocrinology 13 (2025): 178–187. 39870096 10.1016/S2213-8587(24)00313-9

[22] N. A. Marston , F. K. Kamanu , G. E. M. Melloni , et al., “Endothelial Cell‐related Genetic Variants Identify LDL Cholesterol‐sensitive Individuals Who Derive Greater Benefit From Aggressive Lipid Lowering, ” Nature Medicine 31 (2025): 963–969. 10.1038/s41591-025-03533-w PMC13014400 40011692

[23] U. Laufs , R. De Caterina , F. Schiele , et al., “The ACS EuroPath Survey Series: Time Trends in Lipid Management After an Acute Coronary Syndrome, ” European Journal of Preventive Cardiology (2025): zwaf399, 10.1093/eurjpc/zwaf399. 40590247

[24] P. Gaba , M. L. O'Donoghue , J. Park , et al., “Association Between Achieved Low‐density Lipoprotein Cholesterol Levels and Long‐term Cardiovascular and Safety Outcomes: An Analysis of FOURIER‐OLE, ” Circulation 147 (2023): 1192–1203. 36779348 10.1161/CIRCULATIONAHA.122.063399

[25] G. G. Schwartz , M. Szarek , D. L. Bhatt , et al., “Transiently Achieved Very Low LDL‐cholesterol Levels by Statin and Alirocumab After Acute Coronary Syndrome Are Associated With Cardiovascular Risk Reduction: The ODYSSEY OUTCOMES Trial, ” European Heart Journal 44 (2023): 1408–1417. 36879424 10.1093/eurheartj/ehad144 PMC10119028

[26] E. Hagström , P. G. Steg , M. Szarek , et al., “Apolipoprotein B, Residual Cardiovascular Risk After Acute Coronary Syndrome, and Effects of alirocumab, ” Circulation 146 (2022): 657–672. 35770629 10.1161/CIRCULATIONAHA.121.057807 PMC9422774

[27] D. Gaudet , J. L. López‐Sendón , M. Averna , et al., “Safety and Efficacy of alirocumab in a Real‐life Setting: The ODYSSEY APPRISE Study, ” European Journal of Preventive Cardiology 28 (2022): 1864–1872. 33624041 10.1093/eurjpc/zwaa097

[28] M. Szarek , E. Reijnders , J. W. Jukema , et al., “Relating Lipoprotein(a) Concentrations to Cardiovascular Event Risk After Acute Coronary Syndrome: A Comparison of 3 Tests, ” Circulation 149 (2024): 192–203. 37632469 10.1161/CIRCULATIONAHA.123.066398 PMC10782942

[29] P. M. Ridker , D. L. Bhatt , A. D. Pradhan , R. J. Glynn , J. G. MacFadyen , and S. E. Nissen , “Inflammation and Cholesterol as Predictors of Cardiovascular Events Among Patients Receiving Statin Therapy: A Collaborative Analysis of Three Randomised Trials, ” Lancet London England 401 (2023): 1293–1301. 36893777 10.1016/S0140-6736(23)00215-5

[30] N. A. A. Azhar , Y. Chua , H. Nawawi , and S. A. Jusoh , “Structural Dynamics of LDL Receptor Interactions With E498A and R499G Variants of PCSK9, ” Journal of Molecular Modeling 31 (2025): 161. 40388017 10.1007/s00894-025-06380-1

[31] Y. Guan , X. Liu , Z. Yang , et al., “PCSK9 promotes LDLR Degradation by Preventing SNX17‐mediated LDLR Recycling, ” Circulation 151 (2025): 1512–1526. 40071387 10.1161/CIRCULATIONAHA.124.072336

[32] A. Canfrán‐Duque , N. Rotllan , X. Zhang , et al., “Macrophage‐Derived 25‐Hydroxycholesterol Promotes Vascular Inflammation, Atherogenesis, and Lesion Remodeling, ” Circulation 147 (2023): 388–408. 36416142 10.1161/CIRCULATIONAHA.122.059062 PMC9892282

[33] R. I. Jaén , A. Povo‐Retana , C. Rosales‐Mendoza , et al., “Functional Crosstalk Between PCSK9 Internalization and Pro‐inflammatory Activation in human Macrophages: Role of Reactive Oxygen Species Release, ” International Journal of Molecular Sciences 23 (2022): 9114. 36012389 10.3390/ijms23169114 PMC9409451

[34] S. Katsuki , P. K. Jha , A. Lupieri , et al., “Proprotein Convertase Subtilisin/Kexin 9 (PCSK9) Promotes Macrophage Activation via LDL Receptor‐Independent Mechanisms, ” Circulation Research 131 (2022): 873–889. 36263780 10.1161/CIRCRESAHA.121.320056 PMC9973449

[35] D. Gomes , S. Wang , L. Goodspeed , et al., “Comparison Between Genetic and Pharmaceutical Disruption of Ldlr Expression for the Development of Atherosclerosis, ” Journal of Lipid Research 63 (2022): 100174. 35101425 10.1016/j.jlr.2022.100174 PMC8953673

[36] Z. Peng , S. Lv , H. Chen , et al., “Disruption of PCSK9 Suppresses Inflammation and Attenuates Abdominal Aortic Aneurysm Formation, ” Arteriosclerosis, Thrombosis, and Vascular Biology 45 (2025): e1–e14. 39588646 10.1161/ATVBAHA.123.320391

[37] P. Xie , H. Luo , W. Pei , et al., “Saponins Derived From *Gynostemma Pentaphyllum* Regulate Triglyceride and Cholesterol Metabolism and the Mechanisms: A Review, ” Journal of Ethnopharmacology 319 (2024): 117186. 37722515 10.1016/j.jep.2023.117186

[38] F. Teng , X.‐W. Li , M. Li , et al., “components and Lipid‐lowering Effect of Total Saponins From Underground Part of Gynostemma Pentaphyllum], ” Zhongguo Zhong Yao Za Zhi Zhongguo Zhongyao Zazhi China J Chin Mater Medica 47 (2022): 5022–5031. 10.19540/j.cnki.cjcmm.20220613.701 36164912

[39] Q. Chen , F. Wu , and Y. Zhao , Effects of Gynostemma Pentaphyllum on Hypolipidemic and the Mechanism of Network Pharmacology and Molecular Docking in Treating Hyperlipidaemia. Preprint at, 10.21203/rs.3.rs-1798888/v1 (2022).

[40] P. Xie , J. Xie , M. Xiao , et al., “Liver Lipidomics Analysis Reveals the Anti‐obesity and Lipid‐lowering Effects of Gypnosides From Heat‐processed *Gynostemma Pentaphyllum* in High‐fat Diet Fed Mice, ” Phytomedicine 115 (2023): 154834. 37094422 10.1016/j.phymed.2023.154834

[41] Y. Zou , Z. Chen , X. Zhang , et al., “Targeting PCSK9 Ameliorates Graft Vascular Disease in Mice by Inhibiting NLRP3 Inflammasome Activation in Vascular Smooth Muscle Cells, ” Frontiers in Immunology 13 (2022): 894789. 35720337 10.3389/fimmu.2022.894789 PMC9204514

[42] Y. Wang , D. Fang , Q. Yang , et al., “Interactions Between PCSK9 and NLRP3 Inflammasome Signaling in Atherosclerosis, ” Frontiers in Immunology 14 (2023): 1126823. 36911736 10.3389/fimmu.2023.1126823 PMC9992811

[43] Y. X. Zhang , W. J. Cliff , G. I. Schoefl , and G. Higgins , “Coronary C‐reactive Protein Distribution: Its Relation to Development of Atherosclerosis, ” Atherosclerosis 145 (1999): 375–379. 10488966 10.1016/s0021-9150(99)00105-7

[44] A. A. Momtazi‐Borojeni , S. Sabouri‐Rad , A. M. Gotto , et al., “PCSK9 and Inflammation: A Review of Experimental and Clinical Evidence, ” European Heart Journal ‐ Cardiovascular Pharmacotherapy 5 (2019): 237–245. 31236571 10.1093/ehjcvp/pvz022

[45] E. Punch , J. Klein , P. Diaba‐Nuhoho , H. Morawietz , and M. Garelnabi , “Effects of PCSK9 Targeting: Alleviating Oxidation, Inflammation, and Atherosclerosis, ” Journal of the American Heart Association 11 (2022): e023328. 35048716 10.1161/JAHA.121.023328 PMC9238481

[46] M. K. Jain and P. M. Ridker , “Anti‐inflammatory Effects of Statins: Clinical Evidence and Basic Mechanisms, ” Nature Reviews Drug Discovery 4 (2005): 977–987. 16341063 10.1038/nrd1901

[47] Z. Tang , L. Jiang , J. Peng , et al., “PCSK9 siRNA Suppresses the Inflammatory Response Induced by oxLDL Through Inhibition of NF‐κB Activation in THP‐1‐derived Macrophages, ” International Journal of Molecular Medicine 30 (2012): 931–938. 22825241 10.3892/ijmm.2012.1072

[48] S. Shin , H. Park , H. Chang , et al., “Impact of Intensive LDL Cholesterol Lowering on Coronary Artery Atherosclerosis Progression: A Serial CT Angiography Study, ” JACC Cardiovasc Imaging 10 (2017): 437–446. 27771404 10.1016/j.jcmg.2016.04.013

[49] S. Lee , H. Chang , J. M. Sung , et al., “Effects of Statins on Coronary Atherosclerotic Plaques: The PARADIGM Study, ” JACC Cardiovasc Imaging 11 (2018): 1475–1484. 29909109 10.1016/j.jcmg.2018.04.015

[50] J. M. Smit , A. R. van Rosendael , M. El Mahdiui , et al., “Impact of Clinical Characteristics and Statins on Coronary Plaque Progression by Serial Computed Tomography Angiography, ” Circulation: Cardiovascular Imaging 13 (2020): e009750. 32160786 10.1161/CIRCIMAGING.119.009750

[51] C. M. Gibson , D. Duffy , M. C. Bahit , et al., “Apolipoprotein a‐I Infusions and Cardiovascular Outcomes in Acute Myocardial Infarction According to Baseline LDL‐cholesterol Levels: The AEGIS‐II Trial, ” European Heart Journal 45 (2024): 5023–5038. 39221651 10.1093/eurheartj/ehae614

[52] L. Barbieri , G. Tumminello , I. Fichtner , et al., “PCSK9 and Coronary Artery Plaque‐new Opportunity or Red Herring?, ” Current Atherosclerosis Reports 26 (2024): 589–602. 39150672 10.1007/s11883-024-01230-6 PMC11393034

[53] C. Landlinger , M. G. Pouwer , C. Juno , et al., “The AT04A Vaccine Against Proprotein Convertase Subtilisin/Kexin Type 9 Reduces Total Cholesterol, Vascular Inflammation, and Atherosclerosis in APOE*3Leiden.CETP Mice, ” European Heart Journal 38 (2017): 2499–2507. 28637178 10.1093/eurheartj/ehx260 PMC5837708

[54] Q. Zhang , M. Miao , S. Cao , et al., “PCSK9 promotes Vascular Neointimal Hyperplasia Through Non‐lipid Regulation of Vascular Smooth Muscle Cell Proliferation, Migration, and Autophagy, ” Biochemical and Biophysical Research Communications 742 (2025): 151081. 39632291 10.1016/j.bbrc.2024.151081

[55] R. Xu , T. Li , J. Luo , et al., “increases Vulnerability of Carotid Plaque by Promoting Mitochondrial Dysfunction and Apoptosis of Vascular Smooth Muscle Cells, ” CNS Neuroscience and Therapeutics 30 (2024): e14640. 38402551 10.1111/cns.14640 PMC10894644

[56] T. A. Lagace , “PCSK9 and LDLR Degradation: Regulatory Mechanisms in Circulation and in Cells, ” Current Opinion in Lipidology 25 (2014): 387–393. 25110901 10.1097/MOL.0000000000000114 PMC4166010

[57] J. M. Cheng , R. M. Oemrawsingh , H. M. Garcia‐Garcia , et al., “PCSK9 in Relation to Coronary Plaque Inflammation: Results of the ATHEROREMO‐IVUS Study, ” Atherosclerosis 248 (2016): 117–122. 27015246 10.1016/j.atherosclerosis.2016.03.010

[58] J. P. Ferreira , C. Xhaard , Z. Lamiral , et al., “PCSK9 protein and rs562556 Polymorphism Are Associated With Arterial Plaques in Healthy Middle‐aged Population: The STANISLAS Cohort, ” Journal of the American Heart Association 9 (2020): e014758. 32208829 10.1161/JAHA.119.014758 PMC7428603

[59] L. Saba , T. Saam , H. R. Jäger , et al., “Imaging Biomarkers of Vulnerable Carotid Plaques for Stroke Risk Prediction and Their Potential Clinical Implications, ” Lancet Neurology 18 (2019): 559–572. 30954372 10.1016/S1474-4422(19)30035-3

[60] S. Yasmeen , B. H. Akram , A. H. Hainsworth , and C. Kruuse , “Cyclic Nucleotide Phosphodiesterases (PDEs) and Endothelial Function in Ischaemic Stroke. A Review, ” Cell Signalling 61 (2019): 108–119. 31132399 10.1016/j.cellsig.2019.05.011

[61] E. Rexhaj , S. Bär , R. Soria , et al., “Effects of alirocumab on Endothelial Function and Coronary Atherosclerosis in Myocardial Infarction: A PACMAN‐AMI Randomized Clinical Trial Substudy, ” Atherosclerosis 392 (2024): 117504. 38513436 10.1016/j.atherosclerosis.2024.117504

[62] S. Ac , I. Breder , J. Barreto , et al., “Evolocumab on Top of empagliflozin Improves Endothelial Function of Individuals With Diabetes: Randomized Active‐controlled Trial, ” Cardiovascular Diabetology 21 (2022): 147. 35933413 10.1186/s12933-022-01584-8 PMC9356512

[63] J. Gao , F. Wu , Y. Liu , et al., “Increase of PCSK9 Expression in Diabetes Promotes VEGFR2 Ubiquitination to Inhibit Endothelial Function and Skin Wound Healing, ” Science China Life Sciences 67 (2024): 2635–2649. 39153050 10.1007/s11427-023-2688-8

[64] D. J. McClintick , M. L. O'Donoghue , G. M. De Ferrari , et al., “Long‐term Efficacy of evolocumab in Patients With or Without Multivessel Coronary Disease, ” Journal of the American College of Cardiology 83 (2024): 652–664. 38325990 10.1016/j.jacc.2023.11.029

[65] G. G. Schwartz and R. P. Giugliano , “Proprotein Convertase Subtilisin/Kexin Type 9 Inhibition After Acute Coronary Syndrome or Prior Myocardial Infarction, ” Current Opinion in Lipidology 33 (2022): 147–159. 35695614 10.1097/MOL.0000000000000830

[66] P. Theofilis , A. Papanikolaou , P. K. Vlachakis , et al., “PCSK9 inhibitors and Coronary Atherosclerotic Plaque Modification: A Meta‐analysis, ” European Heart Journal 45 (2024): ehae666.1402.

[67] N. D'Onofrio , F. Prattichizzo , R. Marfella , et al., “SIRT3 mediates the Effects of PCSK9 Inhibitors on Inflammation, Autophagy, and Oxidative Stress in Endothelial Cells, ” Theranostics 13 (2023): 531–542. 36632236 10.7150/thno.80289 PMC9830434

[68] X. Cao , V. W. Y. Wu , Y. Han , et al., “Role of Argininosuccinate Synthase 1 ‐dependent L‐arginine Biosynthesis in the Protective Effect of Endothelial Sirtuin 3 Against Atherosclerosis, ” Advancement of Science 11 (2024): 2307256. 10.1002/advs.202307256 PMC10966544 38233193

[69] Y. Wang , S. Cao , Z. Wang , et al., “PCSK9 affects Vascular Senescence Through the SIRT1 Pathway, ” Experimental Gerontology 201 (2025): 112701. 39921077 10.1016/j.exger.2025.112701

[70] B. Furie and B. C. Furie , “The Molecular Basis of Blood Coagulation, ” Cell 53 (1988): 505–518. 3286010 10.1016/0092-8674(88)90567-3

[71] Thrombosis: a major contributor to global disease burden ISTH steering committee for world thrombosis day | request PDF. Researchgate (2024), 10.1160/TH14-08-0671.

[72] M. U. Puteri , N. U. Azmi , M. Kato , and F. C. Saputri , “PCSK9 promotes Cardiovascular Diseases: Recent Evidence About Its Association With Platelet Activation‐induced Myocardial Infarction, ” Life Basel Switzerland 12 (2022): 190. 35207479 10.3390/life12020190 PMC8875594

[73] M. Puccini , U. Landmesser , and U. Rauch , “Pleiotropic Effects of PCSK9: Focus on Thrombosis and Haemostasis, ” Metabolites 12 (2022): 226. 35323669 10.3390/metabo12030226 PMC8950753

[74] E. P. Navarese , M. Kolodziejczak , M. Winter , et al., “Association of PCSK9 With Platelet Reactivity in Patients With Acute Coronary Syndrome Treated With Prasugrel or Ticagrelor: The PCSK9‐REACT Study, ” International Journal of Cardiology 227 (2017): 644–649. 27810295 10.1016/j.ijcard.2016.10.084

[75] D. Pastori , C. Nocella , A. Farcomeni , et al., “Relationship of PCSK9 and Urinary Thromboxane Excretion to Cardiovascular Events in Patients With Atrial Fibrillation,” Journal of the American College of Cardiology 70, 1455–1462 (2017). 28911508 10.1016/j.jacc.2017.07.743

[76] Z. Qi , L. Hu , J. Zhang , et al., “PCSK9 (proprotein convertase subtilisin/kexin 9) enhances Platelet Activation, Thrombosis, and Myocardial Infarct Expansion by Binding to Platelet CD36,” Circulation 143 (2021): 45–61. 32988222 10.1161/CIRCULATIONAHA.120.046290

[77] K. Kotani and M. Banach , “Lipoprotein(a) and Inhibitors of Proprotein Convertase Subtilisin/Kexin Type 9, ” Journal of Thoracic Disease 9 (2017): E78–E82. 28203441 10.21037/jtd.2017.01.40 PMC5303088

[78] B. Moustafa , D. Oparowski , S. Testai , I. Guman , and G. Trifan , “Efficacy and Safety of PCSK9 Inhibitors for Stroke Prevention: Systematic Review and Meta‐analysis, ” Journal of Stroke and Cerebrovascular Diseases 33 (2024). 10.1016/j.jstrokecerebrovasdis.2024.107633 38336118

[79] F. Agnello , M. S. Mauro , C. Rochira , et al., “PCSK9 inhibitors: Current Status and Emerging Frontiers in Lipid Control, ” Expert Review of Cardiovascular Therapy 22 (2024): 41–58. 37996219 10.1080/14779072.2023.2288169

[80] B. Gencer , N. A. Marston , K. Im , et al., “Efficacy and Safety of Lowering LDL Cholesterol in Older Patients: A Systematic Review and Meta‐analysis of Randomised Controlled Trials, ” Lancet 396 (2020): 1637–1643. 33186535 10.1016/S0140-6736(20)32332-1 PMC8015314

[81] A. Avogaro , R. Buzzetti , R. Candido , et al., “Exploring the Benefits of alirocumab as Lipid‐lowering Therapy in People With Diabetes and Very High Cardiovascular Risk, ” Diabetes Research and Clinical Practice 222 (2025): 112055. 40020784 10.1016/j.diabres.2025.112055

[82] P. Guedeney , G. Giustino , S. Sorrentino , et al., “Efficacy and Safety of Alirocumab and Evolocumab: A Systematic Review and Meta‐analysis of Randomized Controlled Trials, ” European Heart Journal 43 (2022): e17–e25. 31270529 10.1093/eurheartj/ehz430

[83] M. Xu , Z. Wang , Y. Zhang , et al., “Recaticimab Monotherapy for Nonfamilial Hypercholesterolemia and Mixed Hyperlipemia: The Phase 3 REMAIN‐1 Randomized Trial, ” Journal of the American College of Cardiology 84 (2024): 2026–2036. 39387764 10.1016/j.jacc.2024.07.035

[84] V. Bittner , “Pleiotropic Effects of PCSK9 (proprotein convertase subtilisin/kexin type 9) Inhibitors?, ” Circulation 134 (2016): 1695–1696. 27895023 10.1161/CIRCULATIONAHA.116.023687

[85] E. P. Navarese , M. Kołodziejczak , V. Schulze , et al., “Effects of Proprotein Convertase Subtilisin/Kexin Type 9 Antibodies in Adults With Hypercholesterolemia: A Systematic Review and Meta‐analysis, ” Annals of Internal Medicine 163 (2015): 40–51. 25915661 10.7326/M14-2957

[86] K. Oyama , R. P. Giugliano , M. Tang , et al., “Effect of evolocumab on Acute Arterial Events Across all Vascular territories : Results From the FOURIER Trial, ” European Heart Journal 42 (2021): 4821–4829. 34537830 10.1093/eurheartj/ehab604

[87] M. L. O'Donoghue , R. P. Giugliano , S. D. Wiviott , et al., “Long‐term Evolocumab in Patients With Established Atherosclerotic Cardiovascular Disease, ” Circulation 146 (2022): 1109–1119. 36031810 10.1161/CIRCULATIONAHA.122.061620

[88] S. Al Said , M. L. O'Donoghue , X. Ran , et al., “Long‐term Lipid Lowering With Evolocumab in Older Individuals, ” Journal of the American College of Cardiology 85 (2025): 504–512. 39909681 10.1016/j.jacc.2024.11.019

[89] S. J. Nicholls , Y. Kataoka , S. E. Nissen , et al., “Effect of evolocumab on Coronary Plaque Phenotype and Burden in Statin‐treated Patients Following Myocardial Infarction, ” JACC Cardiovascular Imaging 15 (2022): 1308–1321. 35431172 10.1016/j.jcmg.2022.03.002

[90] H. Yano , S. Horinaka , and T. Ishimitsu , “Effect of Evolocumab Therapy on Coronary Fibrous Cap Thickness Assessed by Optical Coherence Tomography in Patients With Acute Coronary Syndrome, ” Journal of Cardiology 75 (2020): 289–295. 31495548 10.1016/j.jjcc.2019.08.002

[91] J. Rakocevic , M. Dobric , R. Vucic , et al., “Small Interfering Ribonucleic Acid as Lipid‐Lowering Therapy: Inclisiran in Focus, ” International Journal of Molecular Sciences 24 (2023): 6012. 36983086 10.3390/ijms24066012 PMC10056056

[92] Z. Luo , Z. Huang , F. Sun , et al., “The Clinical Effects of inclisiran, a First‐in‐class LDL‐C Lowering siRNA Therapy, on the LDL‐C Levels in Chinese Patients With Hypercholesterolemia, ” Journal of Clinical Lipidology 17 (2023): 392–400. 37164838 10.1016/j.jacl.2023.04.010

[93] F. Gomez‐Delgado , M. Raya‐Cruz , N. Katsiki , J. Delgado‐Lista , and P. Perez‐Martinez , “Residual Cardiovascular Risk: When Should We Treat It?, ” European Journal of Internal Medicine 120 (2024): 17–24. 37845117 10.1016/j.ejim.2023.10.013

[94] D. M. Lloyd‐Jones , P. B. Morris , C. M. Ballantyne , et al., “2022 ACC Expert Consensus Decision Pathway on the Role of Nonstatin Therapies for LDL‐cholesterol Lowering in the Management of Atherosclerotic Cardiovascular Disease Risk: A Report of the american college of cardiology solution set oversight committee, ” Journal of the American College of Cardiology 80 (2022): 1366–1418. 36031461 10.1016/j.jacc.2022.07.006

[95] C. Gao , B. Zhu , F. Ouyang , et al., “Stepwise Dual Antiplatelet Therapy De‐escalation in Patients After Drug Coated Balloon Angioplasty (REC‐CAGEFREE II): Multicentre, Randomised, Open Label, Assessor Blind, Non‐inferiority Trial, ” Bmj 388 (2025): e082945. 40164448 10.1136/bmj-2024-082945 PMC11955879

[96] A. Dec , A. Niemiec , E. Wojciechowska , et al., “Inclisiran‐a Revolutionary Addition to a Cholesterol‐lowering Therapy, ” International Journal of Molecular Sciences 24 (2023): 6858. 37047830 10.3390/ijms24076858 PMC10095256

[97] L. Räber , Y. Ueki , T. Otsuka , et al., “Effect of alirocumab Added to High‐intensity Statin Therapy on Coronary Atherosclerosis in Patients With Acute Myocardial Infarction: The PACMAN‐AMI Randomized Clinical Trial, ” Jama 327 (2022): 1771–1781. 35368058 10.1001/jama.2022.5218 PMC8978048

[98] P. M. Ridker , J. G. MacFadyen , B. M. Everett , et al., “Relationship of C‐reactive Protein Reduction to Cardiovascular Event Reduction Following Treatment With canakinumab: A Secondary Analysis From the CANTOS Randomised Controlled Trial, ” Lancet London England 391 (2018): 319–328. 29146124 10.1016/S0140-6736(17)32814-3

[99] E. Q. Klug , S. Llerena , L. J. Burgess , et al., “Efficacy and Safety of lerodalcibep in Patients With or at High Risk of Cardiovascular Disease: A Randomized Clinical Trial, ” JAMA Cardiology 9 (2024): 800–807. 38958989 10.1001/jamacardio.2024.1659 PMC11223044

[100] M. G. Silverman , B. A. Ference , K. Im , et al., “Association Between Lowering LDL‐C and Cardiovascular Risk Reduction Among Different Therapeutic Interventions: A Systematic Review and Meta‐analysis, ” Jama 316 (2016): 1289–1297. 27673306 10.1001/jama.2016.13985

[101] I. T. Farmakis , K. C. Christodoulou , L. Hobohm , S. V. Konstantinides , and L. Valerio , “Lipid Lowering for Prevention of Venous Thromboembolism: A Network Meta‐analysis, ” European Heart Journal 45 (2024): 3219–3227. 38874212 10.1093/eurheartj/ehae361

[102] D. Zahger , G. G. Schwartz , W. Du , et al., “Triglyceride Levels, Alirocumab Treatment, and Cardiovascular Outcomes After an Acute Coronary Syndrome, ” Journal of the American College of Cardiology 84 (2024): 994–1006. 39232634 10.1016/j.jacc.2024.06.035

[103] Epigenetic Editing of PCSK9 for a Durable Reduction in Cholesterol. Nature Medicine 31 (2025): 1083–1084. 10.1038/s41591-025-03583-0 40000801

[104] E. Samuel , M. Watford , U. O. Egolum , D. N. Ombengi , H. Ling , and D. W. Cates , “Inclisiran: A First‐in‐class siRNA Therapy for Lowering Low‐density Lipoprotein Cholesterol, ” Annals of Pharmacotherapy 57 (2023): 317–324. 35775133 10.1177/10600280221105169

[105] I. Gouni‐Berthold , J. Schwarz , and H. K. Berthold , “PCSK9 monoclonal Antibodies: New Developments and Their Relevance in a Nucleic Acid‐based Therapy Era, ” Current Atherosclerosis Reports 24 (2022): 779–790. 35900635 10.1007/s11883-022-01053-3 PMC9474394

[106] T. Kosenko , M. Golder , G. Leblond , W. Weng , and T. A. Lagace , “Low Density Lipoprotein Binds to Proprotein Convertase Subtilisin/Kexin Type‐9 (PCSK9) in human Plasma and Inhibits PCSK9‐mediated Low Density Lipoprotein Receptor Degradation*, ” Journal of Biological Chemistry 288 (2013): 8279–8288. 23400816 10.1074/jbc.M112.421370 PMC3605646

[107] G. Lambert , F. Charlton , K. Rye , and D. E. Piper , “Molecular Basis of PCSK9 Function, ” Atherosclerosis 203 (2009): 1–7. 18649882 10.1016/j.atherosclerosis.2008.06.010

[108] L. Da Dalt , A. Baragetti , and G. D. Norata , “Targeting PCSK9 Beyond the Liver: Evidence From Experimental and Clinical Studies, ” Expert Opinion on Therapeutic Targets 29 (2025): 137–157. 40110803 10.1080/14728222.2025.2482545

[109] S. A. Burnap , K. Sattler , R. Pechlaner , et al., “PCSK9 activity Is Potentiated Through HDL Binding, ” Circulation Research 129 (2021): 1039–1053. 34601896 10.1161/CIRCRESAHA.121.319272 PMC8579991

[110] G. G. Schwartz , M. Szarek , V. A. Bittner , et al., “Lipoprotein(a) and Benefit of PCSK9 Inhibition in Patients With Nominally Controlled LDL Cholesterol, ” Journal of the American College of Cardiology 78 (2021): 421–433. 34325831 10.1016/j.jacc.2021.04.102 PMC8822604

[111] Y. Sun , Q. Lv , Y. Guo , et al., “Recaticimab as Add‐on Therapy to Statins for Nonfamilial Hypercholesterolemia: The Randomized, Phase 3 REMAIN‐2 Trial, ” Journal of the American College of Cardiology 84 (2024): 2037–2047. 39505412 10.1016/j.jacc.2024.09.012

[112] S. U. Khan , S. H. Yedlapati , A. N. Lone , et al., “PCSK9 inhibitors and Ezetimibe With or Without Statin Therapy for Cardiovascular Risk Reduction: A Systematic Review and Network Meta‐analysis, ” Bmj 377 (2022): e069116. 35508321 10.1136/bmj-2021-069116

[113] J. I. Morton , D. Liew , G. F. Watts , et al., “Rethinking Cardiovascular Prevention: Cost‐effective Cholesterol Lowering for Statin‐intolerant Patients in Australia and the UK, ” European Journal of Preventive Cardiology 32, no. 13 (2025): 1259–1270. 40112156 10.1093/eurjpc/zwaf114

[114] Q. Hao , B. Aertgeerts , G. Guyatt , et al., “PCSK9 inhibitors and Ezetimibe for the Reduction of Cardiovascular Events: A Clinical Practice Guideline With Risk‐stratified Recommendations, ” Bmj 377 (2022): e069066. 35508320 10.1136/bmj-2021-069066

[115] J. W. Mulder , A. M. Galema‐Boers , and J. E. Roeters van Lennep , “First Clinical Experiences With inclisiran in a Real‐world Setting, ” Journal of Clinical Lipidology 17 (2023): 818–827. 37775462 10.1016/j.jacl.2023.09.005

[116] Y. Tang , S. Li , J. Hu , K. Sun , L. Liu , and D. Xu , “Research Progress on Alternative Non‐classical Mechanisms of PCSK9 in Atherosclerosis in Patients With and Without Diabetes, ” Cardiovascular Diabetology 19 (2020): 33. 32169071 10.1186/s12933-020-01009-4 PMC7071562

[117] J. Golledge , H. S. Lu , and S. Shah , “Proprotein Convertase Subtilisin/Kexin Type 9 as a Drug Target for Abdominal Aortic Aneurysm, ” Current Opinion in Lipidology 35 (2024): 241–247. 39052843 10.1097/MOL.0000000000000945 PMC11387138

[118] S. Kühnast , J. W. A. van der Hoorn , E. J. Pieterman , et al., “Alirocumab Inhibits Atherosclerosis, Improves the Plaque Morphology, and Enhances the Effects of a Statin, ” Journal of Lipid Research 55 (2014): 2103–2112. 25139399 10.1194/jlr.M051326 PMC4174003

[119] S. J. Bernelot Moens , A. E. Neele , J. Kroon , et al., “PCSK9 monoclonal Antibodies Reverse the Pro‐inflammatory Profile of Monocytes in Familial Hypercholesterolaemia, ” European Heart Journal 38 (2017): 1584–1593. 28329114 10.1093/eurheartj/ehx002

[120] A. D. Mazura , A. Ohler , S. E. Storck , et al., “PCSK9 acts as a Key Regulator of Aβ Clearance Across the Blood‐brain Barrier, ” Cellular and Molecular Life Sciences CMLS 79 (2022): 212. 35344086 10.1007/s00018-022-04237-x PMC8960591

[121] C. Liu , J. Chen , H. Chen , et al., “PCSK9 inhibition: From Current Advances to Evolving Future, ” Cells 11 (2022): 2972. 36230934 10.3390/cells11192972 PMC9562883

[122] B. Papotti , M. P. Adorni , C. Marchi , et al., “PCSK9 affects Astrocyte Cholesterol Metabolism and Reduces Neuron Cholesterol Supplying in Vitro: Potential Implications in alzheimer's Disease, ” International Journal of Molecular Sciences 23 (2022): 12192. 36293049 10.3390/ijms232012192 PMC9602670

[123] F. Yan , L. P. Phan , N. T. T. Le , and Y. Jin , “Research Progress on the Protective Mechanism of Proprotein Convertase Subtilisin/Kexin Type 9 Inhibitors on Vascular Endothelium, ” Journal of Clinical Medicine 28 (2024): 142–148.

[124] L. Wang , Z. Wang , J. Shi , et al., “Inhibition of Proprotein Convertase Subtilisin/Kexin Type 9 Attenuates Neuronal Apoptosis Following Focal Cerebral Ischemia via Apolipoprotein E Receptor 2 Downregulation in Hyperlipidemic Mice, ” International Journal of Molecular Medicine 42 (2018): 2098–2106. 30066942 10.3892/ijmm.2018.3797 PMC6108876

[125] Y. Zheng , T. Zhu , G. Li , L. Xu , and Y. Zhang , “PCSK9 inhibitor Protects Against Ischemic Cerebral Injury by Attenuating Inflammation via the GPNMB/CD44 Pathway, ” International Immunopharmacology 126 (2024): 111195. 38048667 10.1016/j.intimp.2023.111195

[126] H. Wang , Q. Wang , J. Wang , et al., “Proprotein Convertase Subtilisin/Kexin Type 9 (PCSK9) Deficiency Is Protective Against Venous Thrombosis in Mice, ” Scientific Reports 7 (2017): 14360. 29084995 10.1038/s41598-017-14307-x PMC5662614

[127] M. Zuin , A. Corsini , C. Dalla Valle , et al., “Role of PCSK9 Inhibitors in Venous Thromboembolism: Current Evidence and Unmet Clinical Needs, ” European Heart Journal ‐ Cardiovascular Pharmacotherapy 10 (2025): 719–724. 39406397 10.1093/ehjcvp/pvae076 PMC11724145

[128] A. Demers , S. Samami , B. Lauzier , et al., “PCSK9 induces CD36 Degradation and Affects Long‐chain Fatty Acid Uptake and Triglyceride Metabolism in Adipocytes and in Mouse Liver, ” Arteriosclerosis, Thrombosis, and Vascular Biology 35 (2015): 2517–2525. 26494228 10.1161/ATVBAHA.115.306032

[129] J. Grune , H. Meyborg , T. Bezhaeva , et al., “PCSK9 regulates the Chemokine Receptor CCR2 on Monocytes, ” Biochemical and Biophysical Research Communications 485 (2017): 312–318. 28232185 10.1016/j.bbrc.2017.02.085

[130] X. Liu , X. Bao , M. Hu , et al., “Inhibition of PCSK9 Potentiates Immune Checkpoint Therapy for Cancer, ” Nature 588 (2020): 693–698. 33177715 10.1038/s41586-020-2911-7 PMC7770056

[131] M. Sharma , M. P. Schlegel , M. S. Afonso , et al., “Regulatory T Cells License Macrophage Pro‐resolving Functions During Atherosclerosis Regression, ” Circulation Research 127 (2020): 335–353. 32336197 10.1161/CIRCRESAHA.119.316461 PMC7367765

[132] W. Yang , Y. Bai , Y. Xiong , et al., “Potentiating the Antitumour Response of CD8+ T Cells by Modulating Cholesterol Metabolism, ” Nature 531 (2016): 651–655. 26982734 10.1038/nature17412 PMC4851431

[133] A. Cordero , M. Rodríguez‐Mañero , L. Fácila , et al., “Prevention of Myocardial Infarction and Stroke With PCSK9 Inhibitors Treatment: A Metanalysis of Recent Randomized Clinical Trials, ” Journal of Diabetes & Metabolic Disorders 19 (2020): 759–765. 33520801 10.1007/s40200-020-00557-6 PMC7843775

[134] G. G. Schwartz , P. G. Steg , M. Szarek , et al., “Alirocumab and Cardiovascular Outcomes After Acute Coronary Syndrome, ” New England Journal of Medicine 379 (2018): 2097–2107. 30403574 10.1056/NEJMoa1801174

[135] J. G. Robinson , M. Farnier , M. Krempf , et al., “Efficacy and Safety of alirocumab in Reducing Lipids and Cardiovascular Events, ” New England Journal of Medicine 372 (2015): 1489–1499. 25773378 10.1056/NEJMoa1501031

[136] P. M. Ridker , J. Tardif , P. Amarenco , et al., “Lipid‐reduction Variability and Antidrug‐antibody Formation With bococizumab, ” New England Journal of Medicine 376 (2017): 1517–1526. 28304227 10.1056/NEJMoa1614062

[137] M. S. Sabatine , R. P. Giugliano , S. D. Wiviott , et al., “Efficacy and Safety of Evolocumab in Reducing Lipids and Cardiovascular Events,” New England Journal of Medicine 372, no. 16 (2015): 1500–1509, https://www.nejm.org/doi/10.1056/NEJMoa1500858. 25773607 10.1056/NEJMoa1500858

[138] A. Zimerman , A. L. F. Kunzler , B. N. Weber , et al., “Intensive Lowering of LDL Cholesterol Levels With evolocumab in Autoimmune or Inflammatory Diseases: An Analysis of the FOURIER Trial, ” Circulation 151, no. 20 (2025): 1467–1476. 40255182 10.1161/CIRCULATIONAHA.124.072756 PMC12088884

[139] J. W. Jukema , L. E. Zijlstra , D. L. Bhatt , et al., “Effect of alirocumab on Stroke in ODYSSEY OUTCOMES, ” Circulation 140 (2019): 2054–2062. 31707788 10.1161/CIRCULATIONAHA.119.043826 PMC6919220

[140] M. J. Koren , M. S. Sabatine , R. P. Giugliano , et al., “Long‐term Efficacy and Safety of evolocumab in Patients With Hypercholesterolemia, ” Journal of the American College of Cardiology 74 (2019): 2132–2146. 31648705 10.1016/j.jacc.2019.08.1024

[141] V. Bittner , M. Bertolet , R. Barraza Felix , et al., “Comprehensive Cardiovascular Risk Factor Control Improves Survival, ” Journal of the American College of Cardiology 66 (2015): 765–773. 26271057 10.1016/j.jacc.2015.06.019 PMC4550809

[142] R. M. Sánchez‐Hernández , D. Ibarretxe , F. Fuentes Jiménez , et al., “Homozygous Familial Hypercholesterolemia in Spain. Data From Registry of the spanish atherosclerosis society, ” Journal of Clinical Endocrinology and Metabolism 110, no. 8 (2024): 2280–2287. 10.1210/clinem/dgae784 39514762

[143] M. J. Wilkinson , P. Bijlani , M. H. Davidson , et al., “Real‐World Effectiveness and Safety of Evinacumab in Children and Adults with Homozygous Familial Hypercholesterolemia: A Multisite US Perspective‐Brief Report, ” Arteriosclerosis, Thrombosis, and Vascular Biology 45 (2025): 1310–1315. 40438928 10.1161/ATVBAHA.124.322364

[144] N. Bansal , R. Katz , C. Robinson‐Cohen , et al., “Absolute Rates of Heart Failure, Coronary Heart Disease, and Stroke in Chronic Kidney Disease: An Analysis of 3 Community‐based Cohort Studies, ” JAMA Cardiology 2 (2017): 314–318. 28002548 10.1001/jamacardio.2016.4652 PMC5832350

[145] F. Raal , R. Durst , R. Bi , et al., “Efficacy, Safety, and Tolerability of inclisiran in Patients With Homozygous Familial Hypercholesterolemia: Results From the ORION‐5 Randomized Clinical Trial, ” Circulation 149 (2024): 354–362. 37850379 10.1161/CIRCULATIONAHA.122.063460 PMC10815002

[146] A. Wiegman , A. L. Peterson , R. A. Hegele , et al., “Efficacy and Safety of inclisiran in Adolescents With Genetically Confirmed Homozygous Familial Hypercholesterolemia: Results From the Double‐blind, Placebo‐controlled Part of the ORION‐13 Randomized Trial, ” Circulation 151 (2025): 1758–1766. 40391436 10.1161/CIRCULATIONAHA.124.073233 PMC12180692

[147] C. Borràs , M. Canyelles , J. Girona , et al., “PCSK9 antibodies Treatment Specifically Enhances the Macrophage‐specific Reverse Cholesterol Transport Pathway in Heterozygous Familial Hypercholesterolemia, ” JACC: Basic to Translational Science 9 (2024): 1195–1210. 39534644 10.1016/j.jacbts.2024.06.008 PMC11551875

[148] Y. Lee , B. Hong , K. H. Yun , et al., “Alternative LDL Cholesterol‐lowering Strategy vs High‐intensity Statins in Atherosclerotic Cardiovascular Disease: A Systematic Review and Individual Patient Data Meta‐analysis, ” JAMA Cardiology 10 (2025): 137–144. 39565634 10.1001/jamacardio.2024.3911 PMC11579890

[149] K. A. Krychtiuk , M. Claeys , B. Gencer , and F. Mach , “In‐hospital Initiation of PCSK9 Inhibitors in ACS: Pros and Cons, ” EuroIntervention 19, no. 4 (2023): e283–e285, https://eurointervention.pcronline.com/article/in‐hospital‐initiation‐of‐pcsk9‐inhibitors‐in‐acs‐pros‐and‐cons. 37458121 10.4244/EIJ-E-23-00014 PMC10333913

[150] Correction to: 2023 ESC Guidelines for the Management of Cardiovascular Disease in Patients With Diabetes: Developed by the Task Force on the Management of Cardiovascular Disease in Patients With Diabetes of the european society of cardiology (ESC). European Heart Journal 45 (2024): 518. 38247449 10.1093/eurheartj/ehad857

[151] J. Los , F. B. Mensink , T. P. J. Jansen , et al., “Functional Improvement of Non‐infarct Related Coronary Artery Stenosis by Extensive LDL‐C Reduction With a PCSK9 Antibody: The FITTER Trial, Microvascular Function Analysis, ” European Heart Journal 45 (2024): ehae666.1288.

[152] E. M. Balke , E. V. Balti , B. Van der Auwera , et al., “Accelerated Progression to Type 1 Diabetes in the Presence of HLA‐A*24 and ‐B*18 Is Restricted to Multiple Islet Autoantibody‐Positive Individuals with Distinct HLA‐DQ and Autoantibody Risk Profiles, ” Diabetes Care 41 (2018): 1076–1083. 29545461 10.2337/dc17-2462

[153] K. K. Ray , E. Bruckert , P. Peronne‐Filardi , et al., “Long‐term Persistence With Evolocumab Treatment and Sustained Reductions in LDL‐cholesterol Levels Over 30 Months: Final Results From the european Observational HEYMANS Study, ” Atherosclerosis 366 (2023): 14–21. 36696747 10.1016/j.atherosclerosis.2023.01.002

[154] K. K. Ray , N. Dhalwani , M. Sibartie , et al., “Low‐density Lipoprotein Cholesterol Levels Exceed the Recommended european Threshold for PCSK9i Initiation: Lessons From the HEYMANS Study, ” European Heart Journal ‐ Quality of Care and Clinical Outcomes 8 (2022): 447–460. 35175350 10.1093/ehjqcco/qcac009 PMC9170569

[155] I. Fernández‐Ruiz , “Alirocumab Induces Plaque Regression, ” Nature Reviews Cardiology 19 (2022): 350. 10.1038/s41569-022-00706-9 35422520

[156] Y. Ueki , J. D. Häner , S. Losdat , et al., “Effect of Alirocumab Added to High‐Intensity Statin on Platelet Reactivity and Noncoding RNAs in Patients With AMI: A Substudy of the PACMAN‐AMI Trial, ” Thrombosis and Haemostasis 124 (2024): 517–527. 37595625 10.1055/a-2156-7872

[157] S. Bär , R. Kavaliauskaite , T. Otsuka , et al., “Impact of alirocumab on Plaque Regression and Haemodynamics of Non‐culprit Arteries in Patients With Acute Myocardial Infarction: A Prespecified Substudy of the PACMAN‐AMI Trial, ” EuroIntervention: Journal of EuroPCR in Collaboration with the Working Group on Interventional Cardiology of the European Society of Cardiology 19 (2023): e286–e296. 37341586 10.4244/EIJ-D-23-00201 PMC10333923

[158] X. Sun , S. Bai , H. Wu , T. Wang , and R. Du , “Administration of Evolocumab in Patients With STEMI After Emergency PCI: A Real‐world Cohort Study, ” American Journal of Cardiovascular Drugs 25, no. 4 (2025): 533–545. 39992584 10.1007/s40256-025-00722-3

[159] F. G. Biccirè , R. Kakizaki , K. C. Koskinas , et al., “Lesion‐level Effects of LDL‐C‐lowering Therapy in Patients With Acute Myocardial Infarction: A Post Hoc Analysis of the PACMAN‐AMI Trial, ” JAMA Cardiology 9 (2024): 1082–1092. 39221516 10.1001/jamacardio.2024.3200 PMC11369785

[160] P. M. Ridker , J. Revkin , P. Amarenco , et al., “Cardiovascular Efficacy and Safety of Bococizumab in High‐Risk Patients, ” New England Journal of Medicine 376 (2017): 1527–1539. 28304242 10.1056/NEJMoa1701488

[161] K. K. Ray , F. J. Raal , D. G. Kallend , et al., “Inclisiran and Cardiovascular Events: A Patient‐level Analysis of Phase III Trials, ” European Heart Journal 44 (2023): 129–138. 36331326 10.1093/eurheartj/ehac594 PMC9825807

[162] W. Zhu , Y. Wu , X. Li , et al., “A Stroke Organoids‐multiomics Platform to Study Injury Mechanism and Drug Response, ” Bioactive Materials 44 (2025): 68–81. 10.1016/j.bioactmat.2024.09.038 PMC13051548 41947984

[163] C. Staehr , V. Hinkley , V. V. Matchkov , et al., “Hypoxia and Ischemic Stroke Modify Cerebrovascular Tone by Upregulating Endothelial BK(Ca) Channels‐lessons From Rat, Pig, Mouse, and human, ” Acta Physiologica (Oxford, England) 241 (2025): e70030. 40116175 10.1111/apha.70030 PMC11926774

[164] T. Yoshikawa , Y. Akiyoshi , K. Motokawa , K. Nojiri , and H. Kawaguchi , “Cerebral Angiography and Neurobehavioral Patterns in a Non‐human Primate Middle Cerebral Artery Occlusion Model, ” Vivo Athens Greece 38 (2024): 2245–2253. 10.21873/invivo.13689 PMC11363800 39187365

[165] G. Li , L. Lan , T. He , et al., “Comprehensive Assessment of Ischemic Stroke in Nonhuman Primates: Neuroimaging, Behavioral, and Serum Proteomic Analysis, ” Acs Chemical Neuroscience 15 (2024): 1548–1559. 38527459 10.1021/acschemneuro.3c00826 PMC10996879

[166] M. Salman , S. Ismael , and T. Ishrat , “A Modified Murine Photothrombotic Stroke Model: A Minimally Invasive and Reproducible Cortical and Sub‐cortical Infarct Volume and Long‐term Deficits, ” Experimental Brain Research 241 (2023): 2487–2497. 37656197 10.1007/s00221-023-06696-5

[167] F. Tremblay , Q. Xiong , S. S. Shah , et al., “A Potent Epigenetic Editor Targeting human PCSK9 for Durable Reduction of Low‐density Lipoprotein Cholesterol Levels, ” Nature Medicine 31, no. 4 (2025): 1329–1338. 10.1038/s41591-025-03508-x PMC12003160 39930141

[168] G. Li , C. Zhang , Y. Li , et al., “Optogenetic Vagal Nerve Stimulation Attenuates Heart Failure by Limiting the Generation of Monocyte‐derived Inflammatory CCRL2+ Macrophages, ” Immunity 58, no. 7 (2025): 1847–1861. 40580954 10.1016/j.immuni.2025.06.003

[169] Y. Zhang , H. Zhang , M. Jiang , et al., “Neuroprotection on Ischemic Brain Injury by Mg2+/H2 Released From Endovascular Mg Implant, ” Bioactive Materials 42 (2024): 124–139. 39280580 10.1016/j.bioactmat.2024.08.019 PMC11402188

[170] K. K. Ray , E. Oru , R. S. Rosenson , et al., “Durability and Efficacy of solbinsiran, a GalNAc‐conjugated siRNA Targeting ANGPTL3, in Adults With Mixed Dyslipidaemia (PROLONG‐ANG3): a Double‐blind, Randomised, Placebo‐controlled, Phase 2 Trial, ” Lancet London England 405 (2025): 1594–1607. 40179932 10.1016/S0140-6736(25)00507-0

[171] K. K. Ray , H. Linnebjerg , L. F. Michael , et al., “Effect of ANGPTL3 Inhibition With Solbinsiran in Preclinical and Early human Studies, ” Journal of the American College of Cardiology 85 (2025): 1803–1818. 40158211 10.1016/j.jacc.2025.03.005

[172] M. Kerneis , F. Cosentino , R. Ferrari , et al., “Impact of Chronic Coronary Syndromes on Cardiovascular Hospitalization and Mortality: The ESC‐EORP CICD‐LT Registry, ” European Journal of Preventive Cardiology 29, no. 15 (2022): 1945–1954. 35653582 10.1093/eurjpc/zwac089

[173] A. M. Cao Zhang , E. Ziogos , T. Harb , G. Gerstenblith , and T. M. Leucker , “Emerging Clinical Role of Proprotein Convertase Subtilisin/Kexin Type 9 Inhibition‐part Two: Current and Emerging Concepts in the Clinical Use of PCSK9 Inhibition, ” European Journal of Clinical Investigation 54 (2024): e14272. 38924090 10.1111/eci.14272

[174] H. D. White , G. G. Schwartz , M. Szarek , et al., “Alirocumab After Acute Coronary Syndrome in Patients With a History of Heart Failure, ” European Heart Journal 43 (2022): 1554–1565. 34922353 10.1093/eurheartj/ehab804 PMC9020985

[175] S. Giordano , J. Ielapi , N. Salerno , et al., “Rationale for Early Administration of PCSK9 Inhibitors in Acute Coronary Syndrome, ” Reviews in Cardiovascular Medicine 25 (2024): 374. 39484117 10.31083/j.rcm2510374 PMC11522761

[176] J. M. Kraaijenhof , N. S. Nurmohamed , A. T. Nordestgaard , et al., “Low‐density Lipoprotein Cholesterol, C‐reactive Protein, and Lipoprotein(a) Universal One‐time Screening in Primary Prevention: The EPIC‐norfolk Study, ” European Heart Journal (2025): ehaf209, 10.1093/eurheartj/ehaf209. PMC12517748 40167249

[177] A. M. Small , A. Pournamdari , G. E. Melloni , et al., “Lipoprotein(a), C‐reactive Protein, and Cardiovascular Risk in Primary and Secondary Prevention Populations, ” JAMA Cardiology 9 (2024): 385–391. 38353970 10.1001/jamacardio.2023.5605 PMC10867772

[178] R. C. Hoogeveen , M. R. Diffenderfer , E. Lim , et al., “Lipoprotein(a) and Risk of Incident Atherosclerotic Cardiovascular Disease: Impact of High‐sensitivity C‐reactive Protein and Risk Variability Among human Clinical Subgroups, ” Nutrients 17 (2025): 1324. 40284189 10.3390/nu17081324 PMC12030245

[179] J. Liao , M. Qiu , X. Su , et al., “The Residual Risk of Inflammation and Remnant Cholesterol in Acute Coronary Syndrome Patients on statin Treatment Undergoing Percutaneous Coronary Intervention, ” Lipids in Health and Disease 23 (2024): 172. 38849939 10.1186/s12944-024-02156-3 PMC11157837

[180] H. Zhang , C. Zhang , Y. Zhang , et al., “The Role of Residual Inflammatory Risk and LDL Cholesterol in Patients With in‐stent Restenosis Undergoing Percutaneous Coronary Intervention, ” Journal of Clinical Lipidology 18 (2024): e746–e755. 39278780 10.1016/j.jacl.2024.05.009

[181] J. Li , K. Yan , P. Zhu , et al., “LDL‐C and hs‐CRP Jointly Modify the Effect of lp(a) on 5‐year Death in Patients With Percutaneous Coronary Intervention, ” Clinical Cardiology 47 (2024): e70025. 39428896 10.1002/clc.70025 PMC11491544

[182] G. Zeng , C. Zhang , Y. Song , et al., “The Potential Impact of Inflammation on the Lipid Paradox in Patients With Acute Myocardial Infarction: A Multicenter Study, ” BMC Medicine [Electronic Resource] 22 (2024): 599. 39710711 10.1186/s12916-024-03823-z PMC11664818

[183] P. M. Ridker , L. Lei , M. J. Louie , et al., “Inflammation and Cholesterol as Predictors of Cardiovascular Events Among 13 970 Contemporary High‐risk Patients With Statin Intolerance, ” Circulation 149 (2024): 28–35. 37929602 10.1161/CIRCULATIONAHA.123.066213 PMC10752259

[184] J. Kim , J. S. Lee , H. Kim , et al., “Differential Impacts of Admission LDL‐cholesterol on Early Vascular Outcomes by Ischemic Stroke Subtypes, ” Journal of Clinical Lipidology 18 (2024): e207–e217. 38101971 10.1016/j.jacl.2023.11.012

[185] Writing Committee , K. K. Birtcher , L. A. Allen , et al., “2022 ACC Expert Consensus Decision Pathway for Integrating Atherosclerotic Cardiovascular Disease and Multimorbidity Treatment: A Framework for Pragmatic, Patient‐centered Care, ” Journal of the American College of Cardiology 81 (2023): 292–317. 36307329 10.1016/j.jacc.2022.08.754

[186] R. Vergallo and C. Patrono , “The PACMAN‐AMI Trial: Game Over for the “Vulnerable Plaque”, ” European Heart Journal 43 (2022): 2179–2180. 35467710 10.1093/eurheartj/ehac222

[187] R. Marfella , F. Prattichizzo , C. Sardu , et al., “Evidence of an Anti‐inflammatory Effect of PCSK9 Inhibitors Within the human Atherosclerotic Plaque, ” Atherosclerosis 378 (2023): 117180. 37422356 10.1016/j.atherosclerosis.2023.06.971

[188] H. Uehara , T. Kajiya , M. Abe , M. Nakata , S. Hosogi , and S. Ueda , “Early and Short‐term Use of Proprotein Convertase Anti‐subtilisin‐kexin Type 9 Inhibitors on Coronary Plaque Stability in Acute Coronary Syndrome, ” European Heart Journal ‐ Open 4 (2024): oeae055. 39131906 10.1093/ehjopen/oeae055 PMC11316204

[189] G. Di Giovanni , Y. Kataoka , K. Bubb , A. J. Nelson , and S. J. Nicholls , “Impact of Lipid Lowering on Coronary Atherosclerosis Moving From the Lumen to the Artery Wall, ” Atherosclerosis 367 (2023): 8–14. 36716526 10.1016/j.atherosclerosis.2023.01.017

[190] F. G. Biccirè , J. Häner , S. Losdat , et al., “Concomitant Coronary Atheroma Regression and Stabilization in Response to Lipid‐lowering Therapy, ” Journal of the American College of Cardiology 82 (2023): 1737–1747. 37640248 10.1016/j.jacc.2023.08.019

[191] L. Pérez de Isla , J. L. Díaz‐Díaz , M. J. Romero , et al., “Alirocumab and Coronary Atherosclerosis in Asymptomatic Patients With Familial Hypercholesterolemia: The ARCHITECT Study, ” Circulation 147 (2023): 1436–1443. 37009731 10.1161/CIRCULATIONAHA.122.062557 PMC10158600

[192] L. Pérez de Isla , J. L. Díaz‐Díaz , M. J. Romero , et al., “Characteristics of Coronary Atherosclerosis Related to Plaque Burden Regression During Treatment With alirocumab: The ARCHITECT Study, ” Circulation: Cardiovascular Imaging 17 (2024): e016206. 38205656 10.1161/CIRCIMAGING.123.016206

[193] G. Di Giovanni and S. J. Nicholls , “Intensive Lipid Lowering Agents and Coronary Atherosclerosis: Insights From Intravascular Imaging, ” American Journal of Preventive Cardiology 11 (2022): 100366. 35856069 10.1016/j.ajpc.2022.100366 PMC9287145

[194] D. Han , E. Tzolos , R. Park , et al., “Effects of Evolocumab on Coronary Plaque Composition and Microcalcification Activity by Coronary PET and CT Angiography, ” JACC Cardiovasc Imaging 18 (2025): 589–599. 40178463 10.1016/j.jcmg.2025.01.005 PMC12058403

[195] E. Ziogos , T. Harb , I. Valenta , et al., “Impact of in‐hospital PCSK9 Inhibition on Myocardial Inflammation After Myocardial Infarction: A Randomized Clinical Trial, ” JACC: Basic to Translational Science 10 (2025): 709–720. 40439630 10.1016/j.jacbts.2025.03.010 PMC12230458

[196] A. E. Lima , G. Vilas Boas , A. L. Carvalho Ferreira , M. Benitez Gonzalez , and C. Guida , “PCSK9 inhibitors Modify Plaque and Reduce Plaque Progression: A Systematic Review and Meta‐analysis of Randomized Controlled Trials, ” Circulation (2023).

[197] Z. Li , L. Guo , Y. An , et al., “Evolocumab Attenuates Myocardial Ischemia/Reperfusion Injury by Blocking PCSK9/LIAS‐mediated Cuproptosis of Cardiomyocytes, ” Basic Research in Cardiology 120 (2025): 301–320. 39930254 10.1007/s00395-025-01100-5

[198] D. Shin , S. Kim , H. Lee , et al., “PCSK9 stimulates Syk, PKCδ, and NF‐κB, Leading to Atherosclerosis Progression Independently of LDL Receptor, ” Nature Communications 15 (2024): 2789. 10.1038/s41467-024-46336-2 PMC10981688 38555386

[199] J. Bi , X. Li , Y. Cheng , et al., “1871‐LB: Impact of PCSK9 Inhibitors on Carotid Plaques in Patients With Diabetes Without Diagnosed ASCVD—A Retrospective Cohort Study,” Diabetes 74 (2025): 1871–LB.

[200] L. Wu , B. Zhang , C. Li , et al., “PCSK9 inhibitors Reduced Early Recurrent Stroke in Patients With Symptomatic Intracranial Atherosclerotic Stenosis, ” Journal of Neurology, Neurosurgery, and Psychiatry 95 (2024): 529–535. 38212060 10.1136/jnnp-2023-332392

[201] M. P. Bonaca , P. Nault , R. P. Giugliano , et al., “Low‐density Lipoprotein Cholesterol Lowering With evolocumab and Outcomes in Patients With Peripheral Artery Disease: Insights From the FOURIER Trial (further cardiovascular outcomes research With PCSK9 inhibition in subjects With elevated risk), ” Circulation 137 (2018): 338–350. 29133605 10.1161/CIRCULATIONAHA.117.032235

[202] R. P. Giugliano , T. R. Pedersen , J. L. Saver , et al., “Stroke Prevention With the PCSK9 (proprotein convertase subtilisin‐kexin type 9) Inhibitor Evolocumab Added to Statin in High‐risk Patients With Stable Atherosclerosis, ” Stroke; A Journal of Cerebral Circulation 51 (2020): 1546–1554. 10.1161/STROKEAHA.119.027759 32312223

[203] Q. Xu , Y. Zhao , N. He , et al., “PCSK9: A Emerging Participant in Heart Failure, ” Biomedicine and Pharmacotherapy 158 (2023): 114106. 36535197 10.1016/j.biopha.2022.114106

[204] W. Zeng , F. Zhou , H. Zhao , et al., “Evaluation of Intensive Statins and Proprotein Convertase Subtilisin/Kexin Type 9 Inhibitors on Intracranial Artery Plaque Stability: A Prospective Single‐arm Study, ” Journal of the American Heart Association 14 (2025): e035651. 39818872 10.1161/JAHA.124.035651 PMC12054507

[205] D. Patriki , S. S. S. Saravi , G. G. Camici , L. Liberale , and J. H. Beer , “PCSK 9: A Link Between Inflammation and Atherosclerosis, ” Current Medicinal Chemistry 29: 251–267. 34238141 10.2174/0929867328666210707192625

[206] S. Pelucchi , L. Da Dalt , G. De Cesare , et al., “Neuronal PCSK9 Regulates Cognitive Performances via the Modulation of ApoER2 Synaptic Localization, ” Pharmacological Research 213 (2025): 107652. 39952371 10.1016/j.phrs.2025.107652

[207] D. L. Alsbrook , M. Di Napoli , K. Bhatia , et al., “Neuroinflammation in Acute Ischemic and Hemorrhagic Stroke, ” Current Neurology and Neuroscience Reports 23 (2023): 407–431. 37395873 10.1007/s11910-023-01282-2 PMC10544736

[208] S. J. Nicholls , “PCSK9 inhibitors and Reduction in Cardiovascular Events: Current Evidence and Future Perspectives, ” Kardiologia Polska 81 (2023): 115–122. 36739653 10.33963/KP.a2023.0030

[209] J. A. Howell , J. Larochelle , R. E. Gunraj , et al., “Effects of Global Ripk2 Genetic Deficiency in Aged Mice Following Experimental Ischemic Stroke, ” Aging Brain 7 (2025): 100135. 40225421 10.1016/j.nbas.2025.100135 PMC11993155

[210] W. He , Y. Li , J. Fan , et al., “Gain‐of‐function PPM1D Mutations Attenuate Ischemic Stroke, ” Cell Death and Differentiation (2025), 10.1038/s41418-025-01523-6. PMC12572320 40399534

[211] Y. Ma , K. Zheng , C. Zhao , et al., “Microglia LILRB4 Upregulation Reduces Brain Damage After Acute Ischemic Stroke by Limiting CD8+ T Cell Recruitment, ” Journal of Neuroinflammation 21 (2024): 214. 39217343 10.1186/s12974-024-03206-4 PMC11366150

[212] I. Sequí‐Domínguez , I. Cavero‐Redondo , C. Álvarez‐Bueno , D. P. Pozuelo‐Carrascosa , S. Nuñez de Arenas‐Arroyo , and V. Martínez‐Vizcaíno , “Accuracy of Pulse Wave Velocity Predicting Cardiovascular and all‐cause Mortality. A Systematic Review and Meta‐analysis, ” Journal of Clinical Medicine 9 (2020): 2080. 32630671 10.3390/jcm9072080 PMC7408852

[213] G. Chang , Y. Hu , Q. Ge , S. Chu , A. Avolio , and J. Zuo , “Arterial Stiffness as a Predictor of the Index of Atherosclerotic Cardiovascular Disease in Hypertensive Patients, ” International Journal of Environmental Research and Public Health 20 (2023): 2832. 36833532 10.3390/ijerph20042832 PMC9957494

[214] S. Misra , P. Singh , S. Sengupta , et al., “Subtyping Strokes Using Blood‐based Protein Biomarkers: A High‐throughput Proteomics and Machine Learning Approach, ” European Journal of Clinical Investigation 55 (2025): e14372. 39655799 10.1111/eci.14372

[215] G. M. De Marchis , P. Krisai , L. Werlen , et al., “Biomarker, Imaging, and Clinical Factors Associated With Overt and Covert Stroke in Patients With Atrial Fibrillation, ” Stroke; A Journal of Cerebral Circulation 54 (2023): 2542–2551. 10.1161/STROKEAHA.123.043302 PMC10519288 37548011

[216] X. Guan , S. Zhu , J. Song , et al., “Microglial CMPK2 Promotes Neuroinflammation and Brain Injury After Ischemic Stroke, ” Cell Reports Medicine 5 (2024): 101522. 38701781 10.1016/j.xcrm.2024.101522 PMC11148565

[217] M. J. Koren , R. B. Vega , N. Agrawal , et al., “An Oral PCSK9 Inhibitor for Treatment of Hypercholesterolemia, ” Journal of the American College of Cardiology 85, no. 21 (2025): 1996–2007. 40167413 10.1016/j.jacc.2025.03.499

[218] C. M. Ballantyne , P. Banka , G. Mendez , et al., “Phase 2b Randomized Trial of the Oral PCSK9 Inhibitor MK‐0616, ” Journal of the American College of Cardiology 81 (2023): 1553–1564. 36889610 10.1016/j.jacc.2023.02.018

[219] S. S. Virani , L. K. Newby , S. V. Arnold , et al., “2023 AHA/ACC/ACCP/ASPC/NLA/PCNA Guideline for the Management of Patients With Chronic Coronary Disease: A Report of the american heart association/american college of cardiology joint committee on Clinical practice Guidelines, ” Circulation 148, no. 9 (2023): e9–e119. 37471501 10.1161/CIR.0000000000001168

[220] A. Greco , S. Finocchiaro , M. Spagnolo , et al., “Lipoprotein(a) as a Pharmacological Target: Premises, Promises, and Prospects, ” Circulation 151 (2025): 400–415. 39928714 10.1161/CIRCULATIONAHA.124.069210

[221] C. Gregorio , F. Rea , F. Ieva , et al., “Flexible Approaches Based on Multistate Models and Microsimulation to Perform Real‐world Cost‐effectiveness Analyses: An Application to Proprotein Convertase Subtilisin‐kexin Type 9 Inhibitors, ” Value in Health: The Journal of the International Society for Pharmacoeconomics and Outcomes Research 27 (2024): 897–906. 38548178 10.1016/j.jval.2024.03.008

[222] G. Barbati , C. Gregorio , A. Scagnetto , C. Indennidate , C. Cappelletto , and A. Di Lenarda , “Effectiveness of PCSK9 Inhibitors: A Target Trial Emulation Framework Based on Real‐world Electronic Health Records, ” PLoS ONE 19 (2024): e0309470. 39173034 10.1371/journal.pone.0309470 PMC11341039

[223] G. Morabito , C. Gregorio , F. Ieva , et al., “Cost‐effectiveness of Single‐pill and Separate‐pill Administration of Antihypertensive Triple Combination Therapy: A Population‐based Microsimulation Study, ” BMC Public Health [Electronic Resource] 24 (2024): 1808. 38971775 10.1186/s12889-024-19346-4 PMC11227134

[224] A. L. Schwartz , S. Kim , A. S. Navathe , and A. Gupta , “Growth of medicare Advantage After Plan Payment Reductions, ” JAMA Health Forum 4 (2023): e231744. 37354538 10.1001/jamahealthforum.2023.1744 PMC10290750

[225] C. Jiang , K. R. Yabroff , R. D. Nipp , et al., “Costs and Access Barriers to ondansetron in the US, ” JAMA Network Open 7 (2024): e2443978. 39509134 10.1001/jamanetworkopen.2024.43978 PMC11544490

[226] A. P. Chung , J. T. Shafrin , S. Vadgama , et al., “Inequalities in CAR T‐cell Therapy Access for US Patients With Relapsed/Refractory DLBCL: A SEER‐medicare Data Analysis, ” Blood Advance 9, no. 18 (2025): 4727–4735. 10.1182/bloodadvances.2024015634 PMC12466227 40378343

[227] Y. Tu , B. Chen , C. Liao , et al., “Inequality in Infrastructure Access and Its Association With Health Disparities, ” Nature Human Behaviour 9, no. 8 (2025): 1669–1682. 10.1038/s41562-025-02208-3 PMC12367552 40404914

[228] L. Vinals , A. Radhakrishnan , and G. Sarri , “Opportunity and Accessibility: An Environmental Scan of Publicly Available Data Repositories to Address Disparities in Healthcare Decision‐making, ” Iternational Journal for Equity in Health 23 (2024): 93. 10.1186/s12939-024-02187-3 PMC11080201 38720282

[229] S. Hayashi , S. Tachibana , T. Maeda , et al., “Real‐world Comparative Study of the Efficacy of janus Kinase Inhibitors in Patients With Rheumatoid Arthritis: The ANSWER Cohort Study, ” Rheumatology Oxford England 63 (2024): 3033–3041. 37924201 10.1093/rheumatology/kead543

